# Synergistic effects in s- and d-block heterometallic compounds: small molecule activation and catalysis

**DOI:** 10.1039/d6sc03347d

**Published:** 2026-08-14

**Authors:** Martin Gawron, Robert Wolf

**Affiliations:** a Institute of Inorganic Chemistry, University of Regensburg 93040 Regensburg Germany robert.wolf@ur.de

## Abstract

Heterometallic compounds involving s-block and d-block metals are increasingly seen as a promising class of catalysts with unique reactivity modes. This perspective summarizes recent advances in key chemical transformations enabled by these heterometallic systems. After a brief overview of their structural features and synergistic reactivity, selected applications in small molecule activation and catalysis are discussed. Examples include hydrogenation, C–X bond activation (X = C, H, O, F, I), alkene isomerization and polymerization, as well as co-polymerization of CO_2_ and epoxides. Structural and mechanistic studies have revealed various synergistic activation modes that depend on the proximity of the metal atoms – achieved through contact ion pairing or ligand design. Collectively, these examples demonstrate that combining s-block and d-block metals is a promising strategy for tuning catalytic behavior and achieving reactivity that surpasses homometallic systems.

## Abstract 2

Ion pairing, ligand preorganization, and surface interactions between s-block and d-block metal atoms enable concerted bond activation pathways, facilitating catalytic applications such as hydrogenation, cross-coupling, and polymerization.

## Graphical abstract

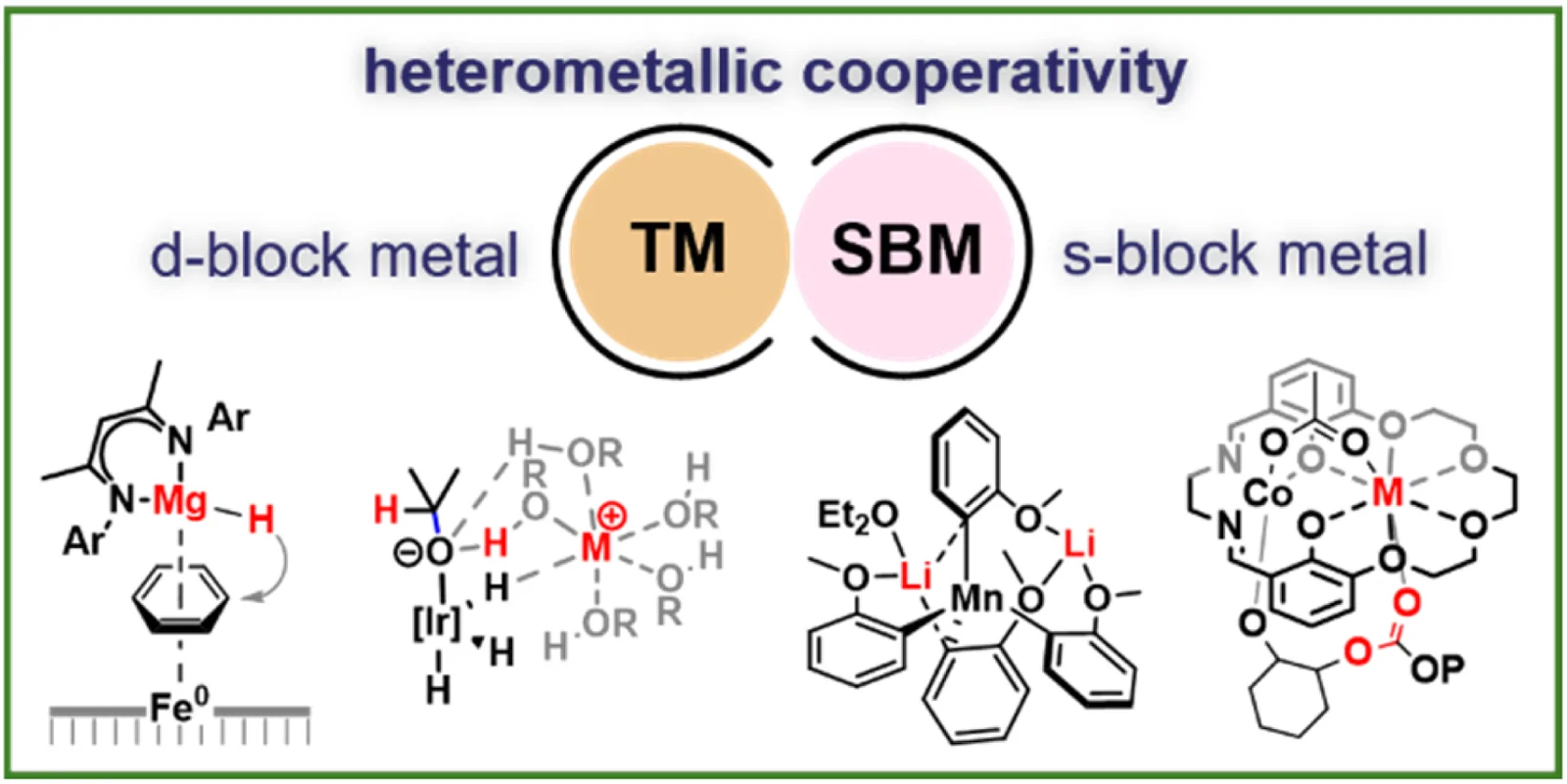

## 1 Introduction

Over the past several decades, heterometallic complexes have become a key focus in coordination chemistry because they enable chemists to combine the complementary properties of different metal atoms within a single molecular structure. Through electronic communication, synergistic substrate binding, or tandem reactivity, interactions between distinct metal sites can generate properties and reactivity patterns inaccessible to monometallic systems.^1–6^

With nearly the entire periodic table at hand, a large array of permutations of chemical elements has been explored (Fig. 1a). Much research has been devoted to designing heterometallic compounds comprising two electronically distinct d-block metal atoms,^2,7–12^ or combinations of d-block and p-block elements,^9,13–25^ for synergistic small molecule activation and catalysis. In parallel, considerable attention is directed toward heterometallic main group compounds that pair an s-block metal with a second s- or p-block element, in which synergistic behavior has only recently been recognized and systematically explored.^26–40^

**Fig. 1:**
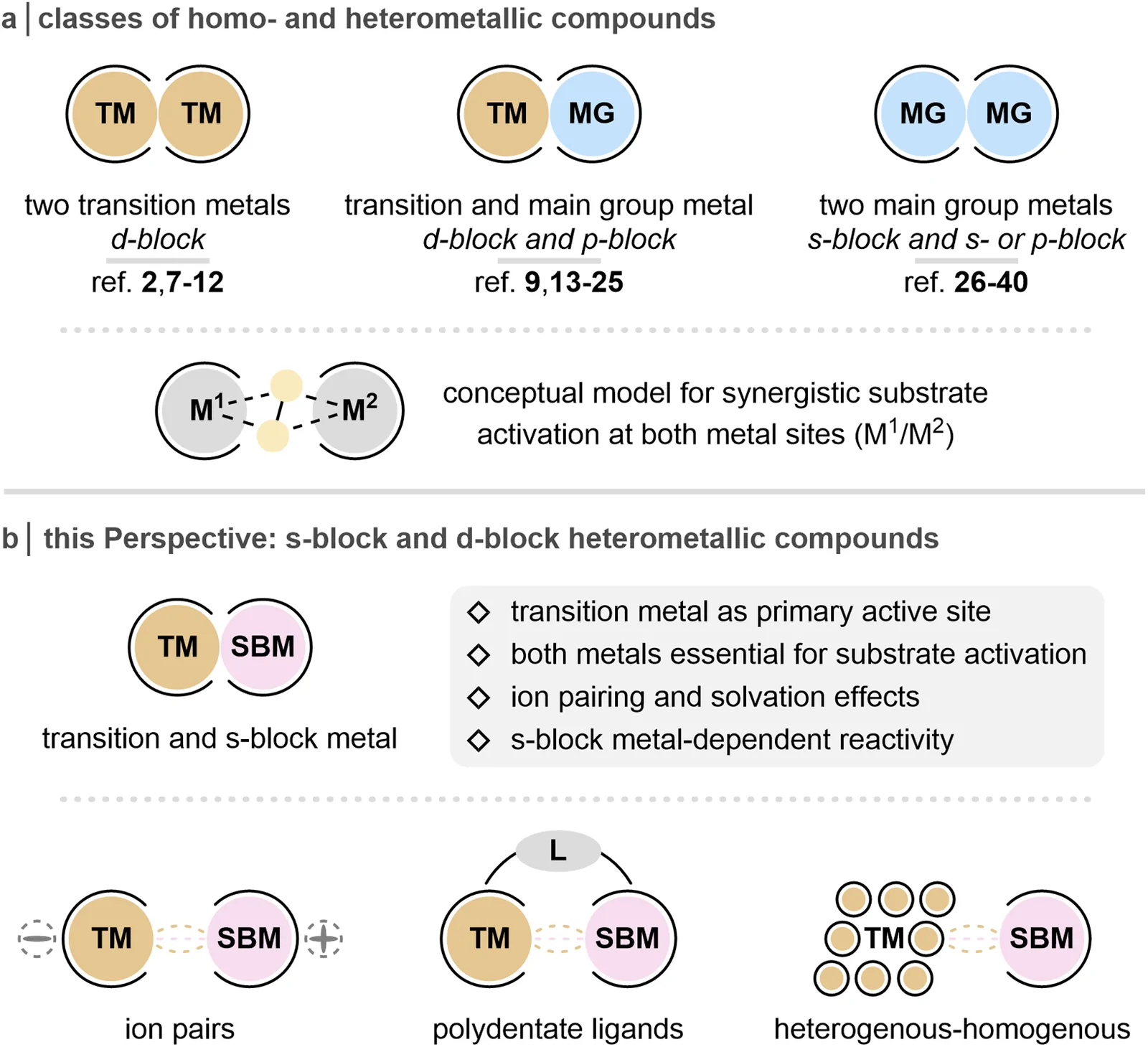
(a) Main classes of homo- and heterometallic compounds and representative references; TM = transition metal, MG = main group element. (b) s- and d-Block heterometallic compounds, distinct structural features and design strategies; SBM = s-block metal.

In contrast, synergistic effects between s-block and d-block metals in transition metalate anions have traditionally received less attention, although the chemistry of transition metalate anions has seen a marked surge over the last two decades.^41–47^ This resurgence is driven in part by the high abundance and low cost of most s-block metals, which motivate their incorporation into catalyst architectures.^48,49^ Recent research has demonstrated that compounds comprising s- and d-block atoms are effective in numerous catalytic processes, including the hydrofunctionalization of unsaturated bonds, C–H functionalization, and polymerization. A key reason for this trend is a growing awareness that s-block metal cations can significantly influence the reactivity of associated transition metal atoms (Fig. 1b). As interest in synergistic catalysis continues to grow, understanding how s-block metals modulate d-block reactivity becomes ever more essential. We aim to clarify the emerging principles and highlight the most promising directions for future catalyst design. This perspective examines key design strategies for heterometallic s-block/d-block metal systems and explores their properties in small molecule activation and catalysis (Fig. 1b). In the first strategy, ion pairing is a decisive factor governing reactivity.^50–52^ Whereas d-block metals engage in covalent bonding,^1,53–55^ s-block and d-block ion pairs predominantly exhibit electrostatic interactions.^56–59^ The strength of the ion pairing is influenced by the size and polarizability of the charged fragments, their relative orientation within the ionic complex, and solvation. A complementary approach for s-block/d-block systems employs polydentate ligand frameworks to achieve intermetallic interactions (Fig. 1b). The ligands provide a fixed spatial relationship between metal atoms that is largely independent of solvation. As a result, they achieve synergistic reactivity with larger structural control. Moreover, the exciting recent developments of heterogeneous–homogeneous catalyst combinations are worth highlighting (Fig. 1b). In these cases, synergistic effects arise at the interface between a metallic TM surface and a homogeneous main group metal species.^60–62^

By examining key transformations, we emphasize the mechanisms at play when d-block and s-block metals work together across these three types. The article is structured into four sections, each dedicated to a major reaction class: hydrogenation, C–X bond activation, isomerization, and polymerization. Special focus is placed on how changing the s-block metal cation influences reactivity, providing a valuable approach for adjusting catalytic performance.

## 2 Cation effects and heterometallic reactivity

### 2.1 Hydrogenation and related reactions

Catalytic hydrogenation with H_2_ gas is an atom-economic and widely used method for reducing organic compounds. Numerous mononuclear transition metal catalysts have been developed to efficiently promote these transformations.^63–70^ Alongside this monometallic toolkit, s-block metal salts of anionic transition metal complexes are increasingly being explored as hydrogenation catalysts.

Kempe and co-workers reported Mn(i) and Fe(i) triazine catalysts bearing a deprotonatable N–H motif for imine and ketone hydrogenation (Fig. 2).^71–73^ Treatment of the neutral precursor complexes [Br_*n*_M(CO)_3−*n*_(PN^triazine^P)] (M = Mn, *n* = 1 or Fe, *n* = 2; PN^triazine^P = 2,6-bis(P^*i*^Pr_2_NH)-4-Ph-triazine) with an excess of KO^*t*^Bu under an H_2_ atmosphere generates potassium salts K_2_[HM(CO)_2_(PN^triazine^P)] (M = Mn or Fe) (Fig. 2a and b), that catalyze the reduction of a wide range of imines and ketones under mild conditions (20 bar H_2_, 50–60 °C, 4–6 h), requiring low catalyst loadings (0.4–1.0 mol%). Various functional groups (*e.g.*, alkene, ketone, nitrile, nitro groups, and halide substituents) were tolerated, and most products were obtained in high yields (>85%) with both Mn(i) and Fe(i) catalysts.

**Fig. 2:**
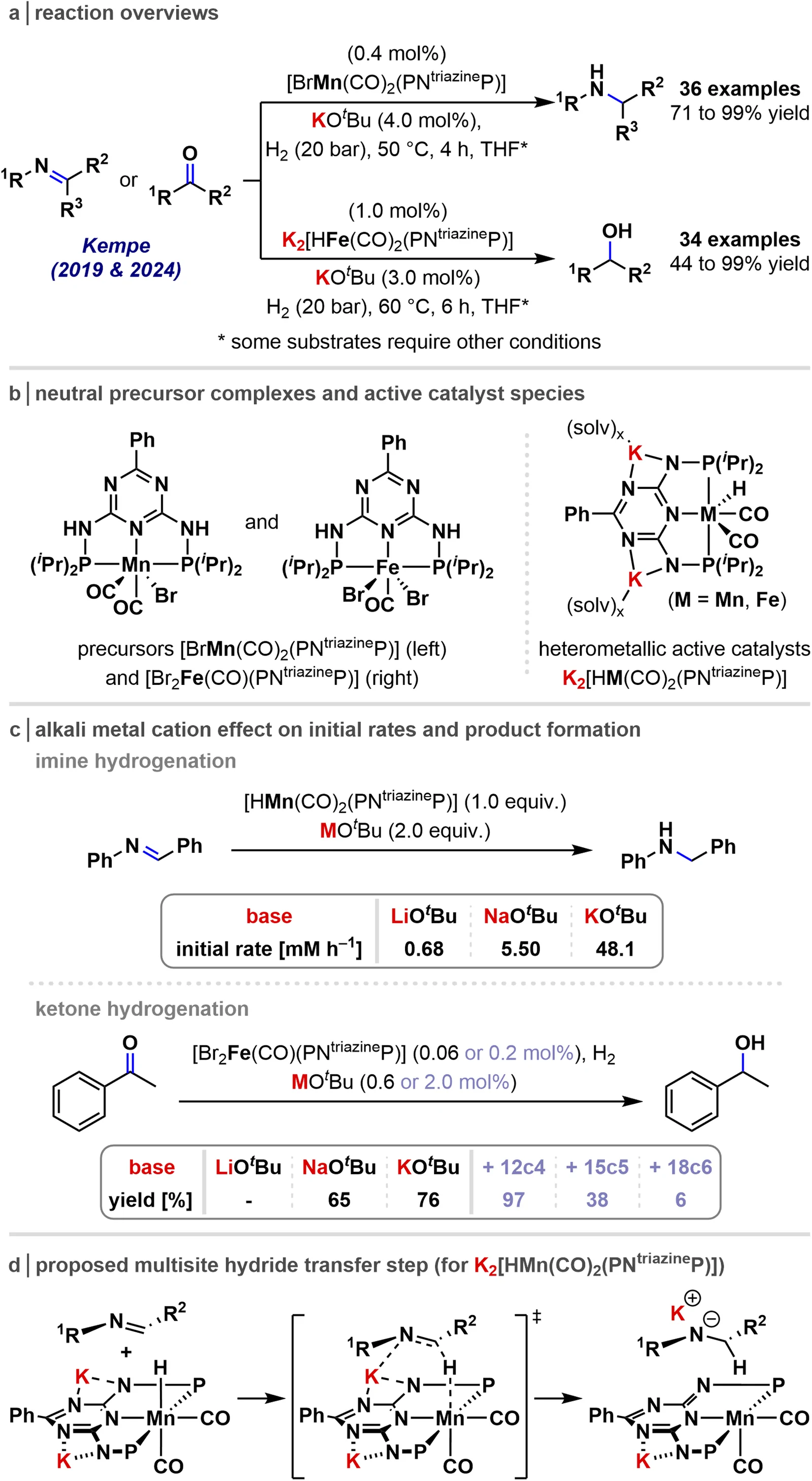
(a) Imine and ketone hydrogenation catalyzed by Mn(i) and Fe(i) triazine complexes. (b) Structures of the pre-catalysts and heterometallic active catalysts. (c) Alkali metal cation influence on reactivity. (d) Proposed rate-determining hydride transfer step (for M = Mn) involving synergistic activation at Mn and K. 12c4 = 12-crown-4. 15c5 = 15-crown-5. 18c6 = 18-crown-6. solv = thf or 12c4.

Mechanistic studies on the role of the alkoxide base in catalyst activation revealed a significant effect of the alkali metal cation on reactivity (Fig. 2c). The potassium salts efficiently hydrogenated the model substrates *N*-benzylideneaniline and acetophenone. The analogous Li^+^ and Na^+^ salts were less active. Kinetic studies of the Mn(i) system revealed that changing the alkoxide base MO^*t*^Bu (M = Li, Na, K) accelerates the hydride transfer to the imine, following the trend Li^+^ < Na^+^ < K^+^ (Fig. 2c).^71^ The substitution of K^+^ by Na^+^ or Li^+^ in the Fe(i) system strongly decreased the catalytic activity.^73^ Moreover, inhibition experiments employing crown ethers as sequestering agents with the most active K_2_[HFe(CO)_2_(PN^triazine^P)] showed that the conversion in the hydrogenation of acetophenone is reduced considerably in the order: 18-crown-6 > 15-crown-5 > 12-crown-4, consistent with their decreasing binding affinity for K^+^. These results indicate that for maximum synergistic reactivity, the alkali metal cation must be positioned close to the transition metal anion. It is proposed that the rate-determining hydride transfer follows an outer-sphere mechanism,^74^ in which both the transition metal and alkali metal play an active role (Fig. 2d for K_2_[HMn(CO)_2_(PN^triazine^P)]). The potassium cation, which is positioned in a triazine ligand cavity, coordinates to the nitrogen atom of the incipient amide during hydride transfer. The resulting short-lived potassium amide then rapidly reacts with HO^*t*^Bu to yield the amine, KO^*t*^Bu, and a monopotassium species, which is subsequently regenerated to the dianionic catalyst K_2_[HMn(CO)_2_(PN^triazine^P)] upon reaction with KO^*t*^Bu and H_2_. In contrast to this ionic pathway, related systems containing an N–H motif form neutral hydride complexes upon treatment with a strong base and H_2_.^75^ In these systems, the hydrogenation of unsaturated substrates (*e.g.*, nitriles) proceeds *via* metal–ligand cooperation through hydride transfer from the transition metal and proton transfer from the ligand-centered N–H site.

Poli, Lledós, Slattery, and co-workers reported the enantioselective hydrogenation of acetophenone with an anionic iridium tetrahydride complex (Fig. 3).^76–78^ The catalytically active ion pair [M(ROH)_*n*_][IrH_4_{(*S*)-P,S^^*i*^Pr^}] (M = Li, Na, K; (*S*)-P,S^^*i*^Pr^ = CpFe[1,2-C_5_H_3_(PPh_2_)(CH_2_S^*i*^Pr)]) is generated *in situ* by reaction of [IrCl(η^4^-cod)]_2_, ferrocene-based ligand (*S*)-P,S^^*i*^Pr^, and alkoxide base MOR (R = ^*i*^Pr or ^*t*^Bu) under catalytic conditions (Fig. 3a and b).^78^ Changing the alkoxide base led to a pronounced change in reactivity, with 1-phenylethanol formation increasing in the order: Li^+^ (20%) < Na^+^ (61%) < K^+^ (90%). This trend could be reproduced for four different ferrocene-based ligands. Furthermore, the addition of 18-crown-6 to the most active potassium system resulted in a drastic reduction in catalytic activity, and no reaction was observed when an ammonium base (NMe_4_OH) was used instead of an alkali metal base. This suggests that an alkali metal cation is crucial for efficient catalysis, and that its proximity to the anion is necessary for synergistic reactivity.

**Fig. 3:**
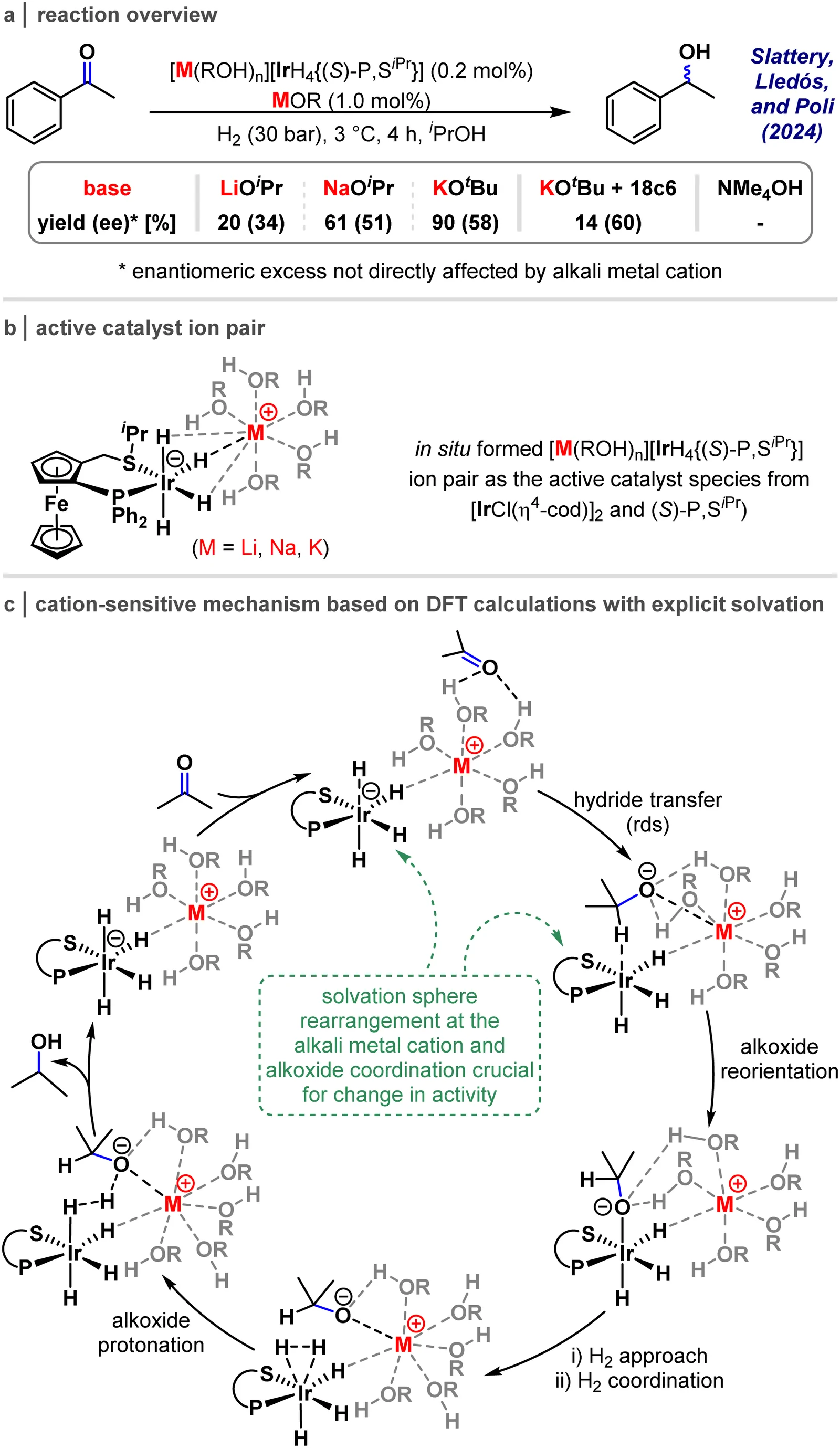
(a) Hydrogenation of acetophenone catalyzed by an alkali metal iridium complex. (b) Proposed structure of the *in situ* formed alkali metal iridium tetrahydride ion pair. (c) Simplified mechanism of the hydrogenation of acetone calculated by DFT. R = Me, ^*i*^Pr.

Detailed DFT calculations on the hydrogenation mechanism involving the catalyst ion pairs showed that the explicit solvation of the alkali metal cation by five alcohol molecules (forming [M(ROH)_5_]^+^; R = Me or ^*i*^Pr) is crucial to reproduce the experimental results (Fig. 3b and c), highlighting the importance of explicit solvation for the accurate modeling of this catalytic reaction. The computational model was designed to accurately represent the first solvation sphere of the alkali metal cation and the overall behavior of the ion pair. The hydrogenation mechanism follows a hydride transfer/protonation sequence, in which the identity of the alkali metal cation directly influences the overall energy barrier (Δ*G*^‡^(K^+^) = 9.8 < Δ*G*^‡^(Na^+^) = 14.1 < Δ*G*^‡^(Li^+^) = 16.6 [kcal mol^−1^]) (Fig. 3c). The acetone substrate initially coordinates to two alcohol molecules in the first solvation sphere of the alkali metal cation, without directly interacting with the cation itself. During the rate-determining hydride transfer, significant reorganization of the first solvation sphere of the cation occurs, which depends on the alkali metal and accounts for the observed changes in the activation barrier. Thus, the cation effect is inherently linked to the coordination sphere rearrangement at the alkali metal cation during the rate-determining hydride transfer. The resulting alkoxide anion subsequently binds to the alkali metal cation, displacing one or more solvent molecules. This change in solvation is most favorable for K^+^, which has a more flexible coordination environment due to its larger ionic radius. Calculations on the tetramethylammonium ion pair ([NMe_4_(ROH)_5_][IrH_4_{(*S*)-P,S^^*i*^Pr^}]) reveal higher activation barriers for this system, supporting the reactivity-enhancing role of the alkali metal cation.

H_2_ activation by mononuclear transition metal complexes typically involves oxidative addition of H_2_ to the metal center, but can also proceed *via* heterometallic cooperation.^11,14,15,79,80^ Most established systems involve either two transition metals or combinations of transition metals with p-block elements, whereas examples of s- and d-block heterometallic systems for H_2_ activation remain rare.^59,81–83^ Xu, Maron, and co-workers synthesized a trinuclear Ni(ii) complex [(^Dipp^nacnac)Mg]_2_Ni(C_2_H_4_)_2_ by reaction of Ni(η^4^-cod)_2_ (cod = 1,5-cyclooctadiene) with (^Dipp^nacnac)MgEt (^Dipp^nacnac = {2,6-^*i*^Pr_2_C_6_H_3_NC(CH_3_)}_2_CH) (Fig. 4).^59^ The molecular structure features a linear Mg–Ni–Mg moiety, with the Ni atom coordinated by two C_2_H_4_ units exhibiting significant metallacyclopropane character. The C and Mg atoms around the Ni center lie in plane, which formally resembles a distorted hexagonal planar coordination environment. Exposure of [(^Dipp^nacnac)Mg]_2_Ni(C_2_H_4_)_2_ to H_2_ (1 bar) led to the formation of [(^Dipp^nacnac)Mg]_2_Ni(µ-H)_4_*via* the intermediate [(^Dipp^nacnac)Mg]_2_Ni(C_2_H_4_)(µ-H)_2_ (Fig. 4b). The structural characterization of [(^Dipp^nacnac)Mg]_2_Ni(µ-H)_4_ identified four covalent Ni–H bonds along with weak Mg–H interactions.

**Fig. 4:**
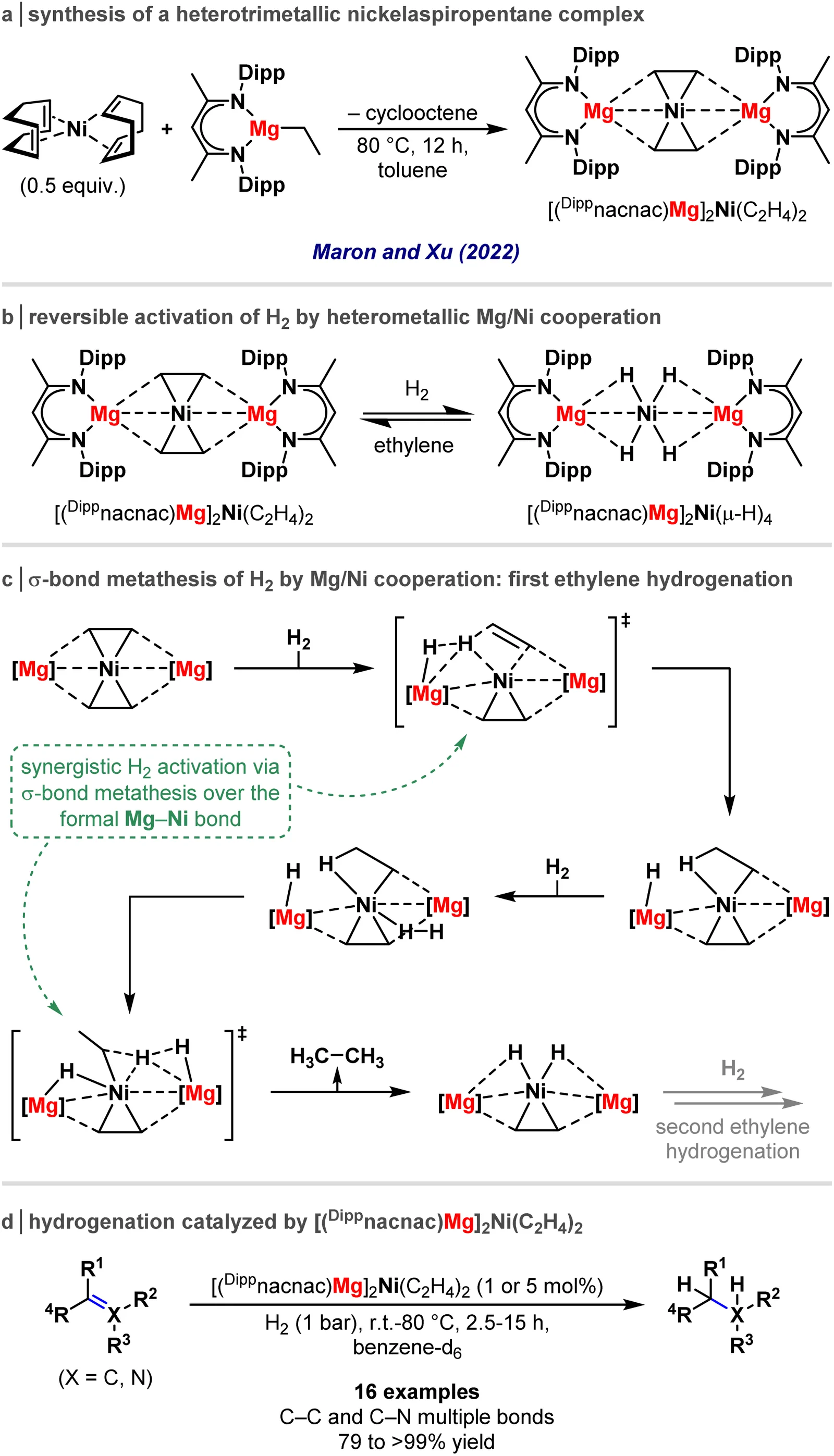
(a) Synthesis of [(^Dipp^nacnac)Mg]_2_Ni(C_2_H_4_)_2_. (b) Reversible activation of H_2_ to give [(^Dipp^nacnac)Mg]_2_Ni(µ-H)_4_. (c) Synergistic σ-bond metathesis of H_2_ over the formal Mg–Ni bond (first ethylene hydrogenation). (c) Catalytic hydrogenation of unsaturated substrates catalyzed by [(^Dipp^nacnac)Mg]_2_Ni(C_2_H_4_)_2_.

DFT calculations of the reaction between [(^Dipp^nacnac)Mg]_2_Ni(C_2_H_4_)_2_ and H_2_ revealed that the cooperation between Mg and Ni enables the stepwise addition of H_2_ without oxidation at the Ni center. In the first step, σ-bond metathesis of H_2_ with the formal Mg–Ni bond occurs, resulting in terminal Mg–H and Ni–H bonds (Fig. 4c). The activation of a second equivalent of H_2_ yields the mixed-ligand complex [(^Dipp^nacnac)Mg]_2_Ni(C_2_H_4_)(µ-H)_2_. Hydrogenation of the remaining ethylene ligand then affords the tetrahydride complex. Due to the facile activation of H_2_, the dimagnesium nickelate served as an efficient pre-catalyst for the hydrogenation of various unsaturated substrates (alkenes, arenes, alkynes, silyl enol ethers, enamines, imines, and quinolines) (Fig. 4d). Using 1 to 5 mol% of [(^Dipp^nacnac)Mg]_2_Ni(C_2_H_4_)_2_ under 1 bar of H_2_, most substrates were hydrogenated in high yields of 79–99%.

[(^Dipp^nacnac)Mg]_2_Ni(C_2_H_4_)_2_ achieved quantitative conversion in silyl enol hydrogenation, whereas conventional monometallic pre-catalysts Ni(η^4^-cod)_2_ and (Ph_3_P)_2_NiCl_2_ had poor catalytic activities. These findings emphasize the crucial role of heterometallic cooperation between the Ni and Mg centers in the activation of H_2_.^81,82^

Crimmin and co-workers showed that a hexagonal planar palladium complex of the composition [(^Dipp^nacnac)Mg]_3_PdH_3,_ can reversibly react with H_2_ to form a tetrahydride species [(^Dipp^nacnac)Mg]_2_Pd(µ-H)_4_ and a magnesium hydride by-product [(^Dipp^nacnac)MgH]_2_.^82^ Similar complexes with PtMg_2_H_4_ and PtZn_2_H_4_ cores were also isolated and characterized through independent methods. DFT results indicated that the (^Dipp^nacnac)Mg fragment promotes H_2_ oxidation at Pd. These insights stimulated the development of a catalytic method for H/D exchange involving magnesium and zinc hydride bonds. Computational studies on a range of heterometallic tetrahydride compounds involving Ni–Pt as well as Mg and Zn suggest these are best described as square planar with only minor metal–metal interactions.^82^ These insights also pertain to the bonding in the magnesium nickelate species reported by Xu, Maron, and co-workers (Fig. 4), warranting further explorations of the bonding situation in these compounds.

Related experimental and theoretical studies on heterometallic hydride complexes bearing (^Dipp^nacnac)MgH and (^Dipp^nacnac)ZnH metalloligands on Pd or Pt revealed a continuum between hexagonal planar and trigonal planar geometries, which is influenced by the group 10 metal as well as the more electropositive s-block or d-block metal (Mg or Zn).^83^ The Zn-containing complex is best described as a tris(σ-zincane) trigonal planar species, whereas the Mg analogues adopt hexagonal planar structures, reflecting the increased ionic character of Mg–H interactions.

Further applications of the dimagnesium nickelates in catalysis include alkene hydrosilylation and H/D exchange (Fig. 5).^84,85^ [(^Dipp^nacnac)Mg]_2_Ni(C_2_H_4_)_2_ effectively catalyzes the hydrosilylation of terminal alkenes with PhSiH_3_ at catalyst loadings of 5–10 mol% and high anti-Markovnikov selectivity (Fig. 5a).^84^ Diverse functional groups were tolerated, including methoxy, amino, boryl, and halogen substituents. The reaction of [(^Dipp^nacnac)Mg]_2_Ni(C_2_H_4_)_2_ with PhSiH_3_ afforded a silyl hydride complex featuring a Ni–Si–H–Mg interaction, closely resembling the structural motif proposed for the σ-bond metathesis of H_2_ (Fig. 4c). This silyl hydride complex catalyzes the hydrosilylation reaction, which likely proceeds through a similar pathway as hydrogenation (Fig. 5b).

**Fig. 5:**
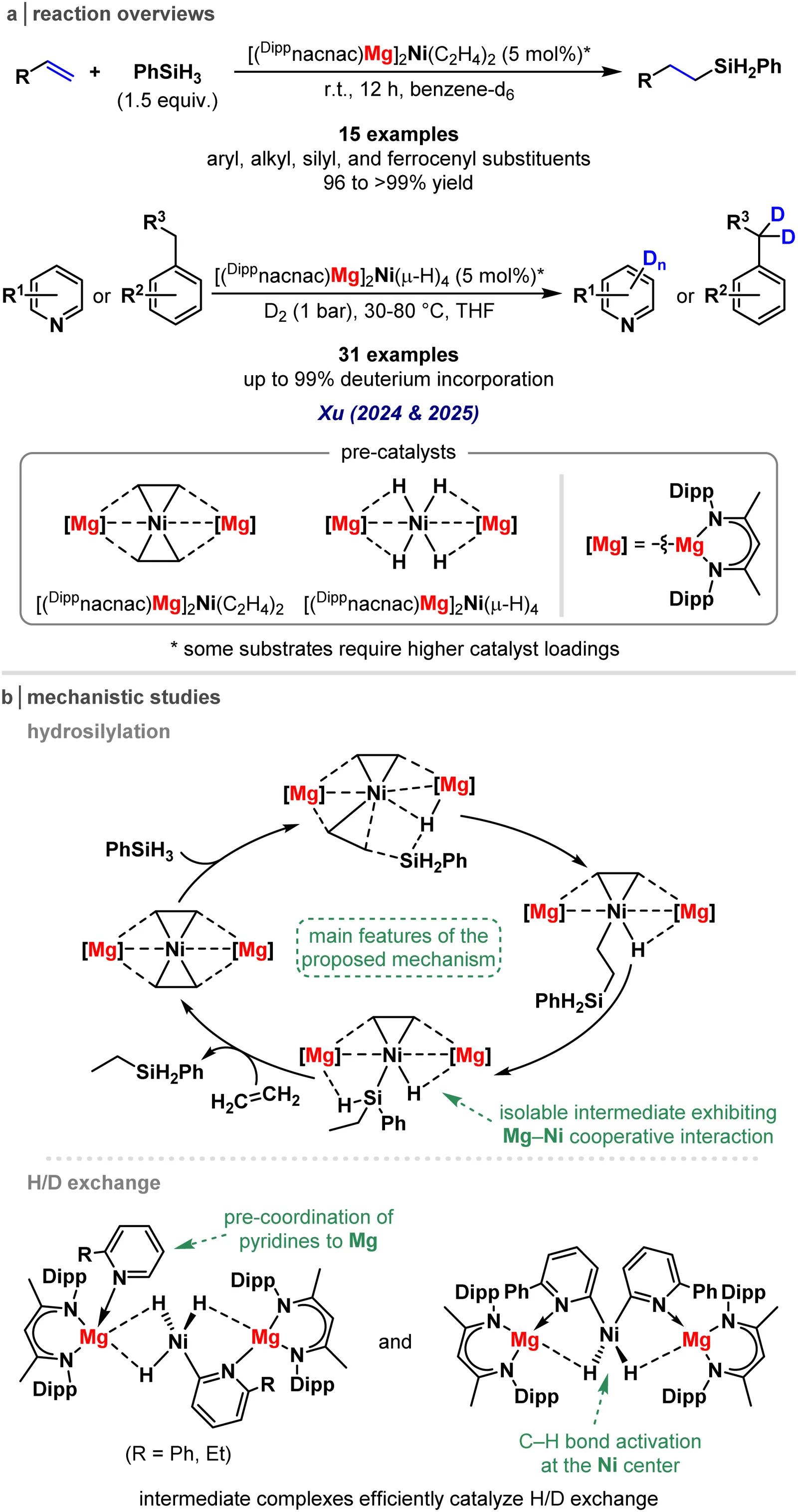
(a) Hydrosilylation of alkenes and H/D exchange of pyridines or arenes catalyzed by [(^Dipp^nacnac)Mg]_2_Ni(C_2_H_4_)_2_ and [(^Dipp^nacnac)Mg]_2_Ni(µ-H)_4_, respectively. (b) Mechanistic studies through isolation of heterometallic intermediates and catalytic reactions starting from these species.

[(^Dipp^nacnac)Mg]_2_Ni(µ-H)_4_ (5 to 10 mol%) also catalyzes the *ortho*-C–H deuteration of pyridines and the benzylic C–H deuteration of alkyl arenes with D_2_ (1 bar) (Fig. 5a), yielding deuterated products with high isotopic incorporation and a good functional group tolerance.^85^ Reactions of [(^Dipp^nacnac)Mg]_2_Ni(µ-H)_4_ with pyridines in a 1 : 2 ratio afforded Mg–Ni–Mg intermediates, in which two pyridine ligands have undergone *ortho*-C–H activation at Ni (Fig. 5b). The resulting complexes efficiently catalyze the deuteration of pyridines, indicating their involvement in the catalytic cycle. Based on the structural features of these intermediates and kinetic isotope effect experiments, a synergistic mechanism was proposed in which the pyridine substrate first coordinates to Mg, followed by *ortho*-C–H bond activation at Ni to form nickel–pyridyl intermediates. These species subsequently react with D_2_*via* σ-bond metathesis to form the deuterated pyridines. Similarly, metal–arene interactions were proposed to play a key role in the benzylic C–H deuteration of alkyl arenes.

A study by Wolf, Rehbein, and co-workers has revealed countercation effects in the hydrogenation of alkenes catalyzed by cobaltate anions (Fig. 6).^86^ A systematic investigation of pre-catalysts of the general structure [Cation][Co(η^4^-cod)_2_] (Cation = K(thf)_0.25_, Na(thf)_1.5_, Li(thf)_2_, (^Dep^nacnac)Mg, and N(^*n*^Bu)_4_, ^Dep^nacnac = {2,6-Et_2_C_6_H_3_NC(CH_3_)}_2_CH) in the alkene hydrogenation revealed the reactivity trend: (^Dep^nacnac)Mg^+^ > Li^+^ > Na^+^ > K^+^ (Fig. 6b). The lack of reactivity observed for the tetra-*n*-butyl-ammonium cobaltate further indicates that a metal cation is essential for hydrogenation activity. The most active pre-catalyst was [(^Dep^nacnac)Mg][Co(η^4^-cod)_2_], which efficiently hydrogenated mono- to tetra-substituted alkenes under mild conditions (2–12 bar H_2_, 30–60 °C, 3–22 h). The homotopic nature of the reaction was confirmed by kinetic analyses, poisoning experiments (Hg test or addition of PMe_3_ or dibenzo[*a*,*e*]cyclooctene) and NMR spectroscopic monitoring.

**Fig. 6:**
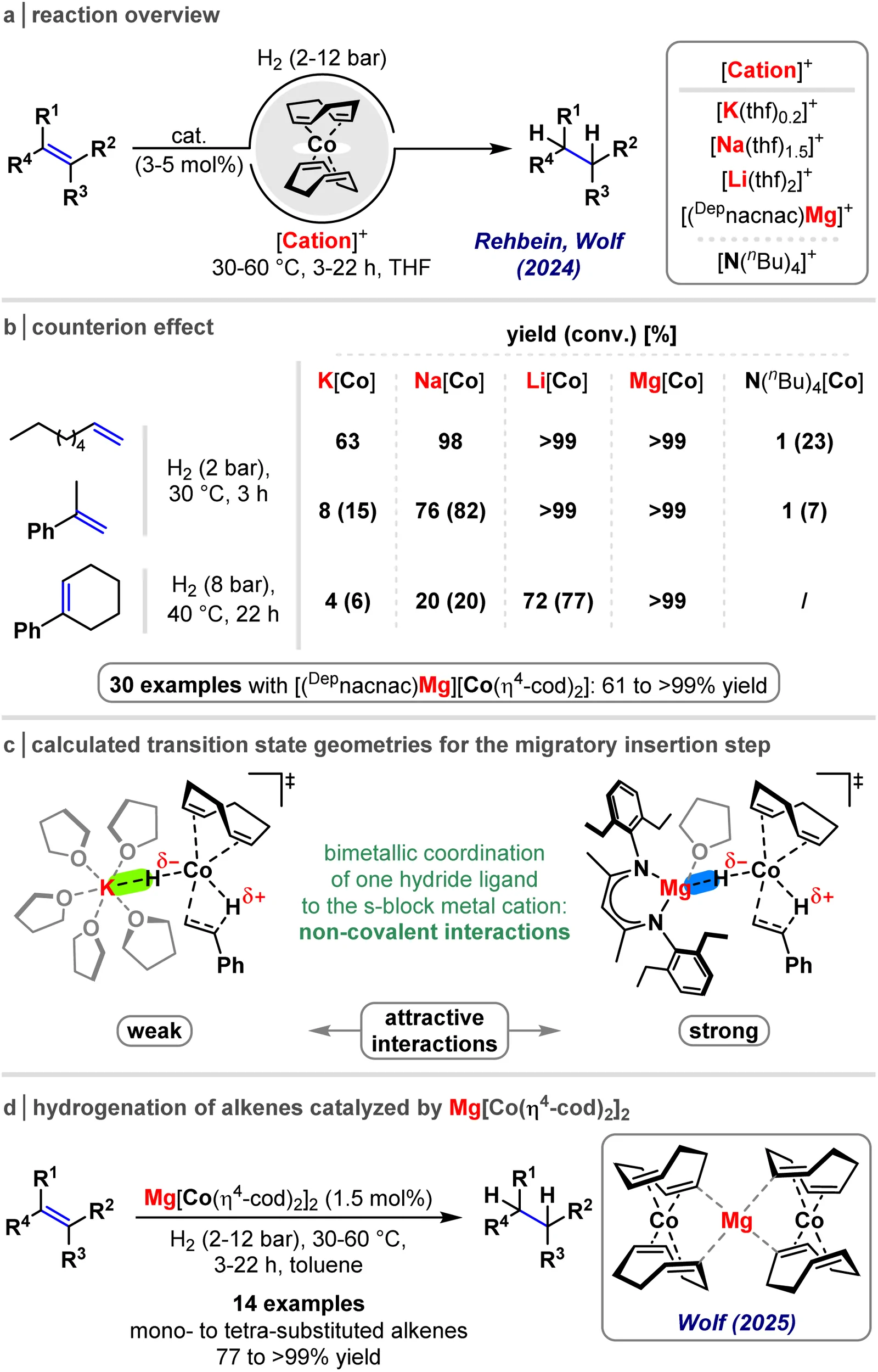
(a) Alkene hydrogenation catalyzed by cobaltate pre-catalysts [Cation][Co(η^4^-cod)_2_] with different cations. (b) Comparative reactivity study showing the cation effect. (c) Proposed [Co–H]⋯M^+^ interaction in the migratory insertion transition state of styrene hydrogenation. (d) Alkene hydrogenation catalyzed by Mg[Co(η^4^-cod)_2_]_2_.

Combined molecular dynamics (MD) simulations and DFT calculations of the styrene hydrogenation mechanism using [M(thf)_*n*_][Co(η^4^-cod)_2_] (M = K(thf)_0.25_, Na(thf)_1.5_, Li(thf)_2_, and (^Dep^nacnac)Mg) suggest that the s-block metal cation coordinates to the hydride ligand ([Co–H]⋯M^+^) during the turnover-limiting migratory insertion step. This interaction is stronger for M^+^ = Li^+^ and (^Dep^nacnac)Mg^+^ than for Na^+^ and K^+^, leading to a stronger Co–H bond polarization and, as a result, lower activation barriers for the Li^+^ and (^Dep^nacnac)Mg^+^ salts (shown for the (^Dep^nacnac)Mg^+^ and K^+^ salts in Fig. 6c). This induces a stronger Co–H bond polarization, which is proposed to improve the catalytic activity. Taken together, these findings demonstrate that countercation coordination can strongly affect the reactivity of metalate anions in hydrogenation and possibly other hydride-mediated reactions.

A similar cation effect was noted for the complex Mg[Co(η^4^-cod)_2_]_2_ prepared by salt metathesis reaction of [K(thf)_0.33_][Co(η^4^-cod)_2_] with 0.5 equivalents of MgCl_2_.^87^ Structural analysis revealed a trinuclear contact ion pair, with no additional ligands supporting the Mg^2+^ cation. The complex efficiently catalyzes the hydrogenation of sterically challenging unfunctionalized alkenes, outperforming the corresponding magnesium β-diketiminate salt [(^Dep^nacnac)Mg][Co(η^4^-cod)_2_]. These results underscore a pronounced counterion effect that is intrinsic to the Mg^2+^ cation and independent of the supporting β-diketiminate ligand.

Harder and co-workers introduced the pairing of a molecular metal species with metal particles as an alternative strategy for heterometallic cooperation that can induce synergistic reactivity at the interface between the metal surface and the dissolved metal species.^61,62^ The combination of (MVS-)Fe^0^ and (MVS-)Ba^0^ powders obtained in multi-gram quantities by metal vapor synthesis (MVS) enabled facile hydrogenation, exhibiting up to three orders of magnitude higher activity than the most active monometallic component (Fig. 7a).^61^ The catalyst is capable of hydrogenating various challenging alkenes, alkynes, imines, and arenes. The hydrogenation of benzene with Fe^0^ or Ba^0^ (10 mol%) did not or only very slowly (>6 d) produce cyclohexane, while the Ba^0^/Fe^0^ mixture (3 mol%) reduced benzene quantitatively within 0.5 h. Mechanistic investigations, including Hg poisoning and recycling experiments, indicate a heterotopic character of the catalytic reaction. However, there is also evidence of soluble Ba species. The surface of a spent Ba^0^/Fe^0^ catalyst is considerably enriched in Ba relative to a fresh catalyst mixture.

**Fig. 7:**
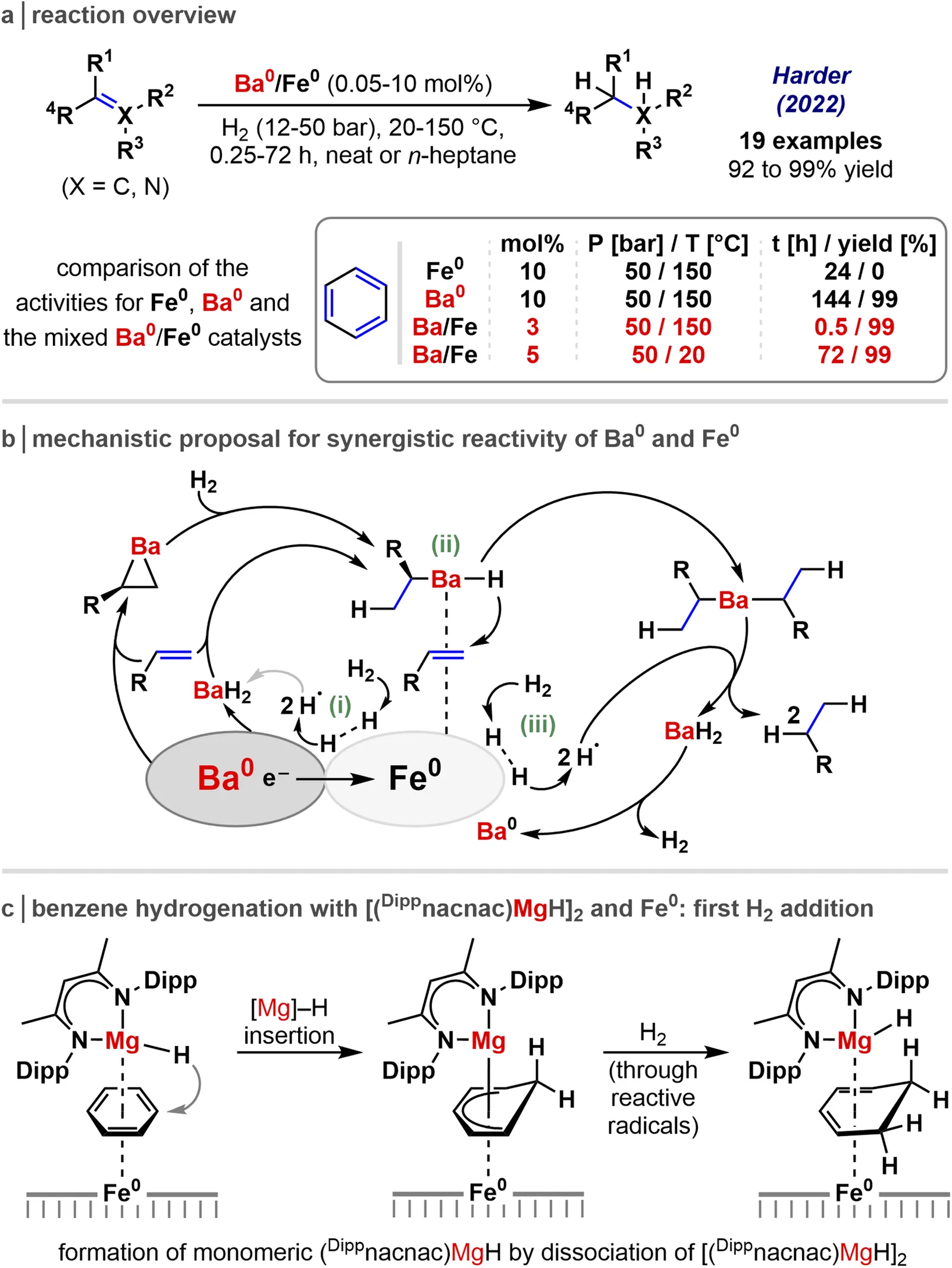
(a) Hydrogenation of unsaturated substrates with Fe^0^, Ba^0^ and Ba^0^/Fe^0^. (b) Mechanistic proposal for the synergistic hydrogenation with Ba^0^ and Fe^0^. (c) Synergistic benzene hydrogenation with soluble [(^Dipp^nacnac)MgH]_2_ and Fe^0^. Fe^0^, Ba^0^ and Ba^0^/Fe^0^ were generated by metal vapor synthesis (MVS).

Based on these insights, a mechanism involving homogenous Ba and heterogeneous Fe^0^ was proposed (Fig. 7b). MVS-activated Ba^0^ first reacts with either H_2_ or an alkene to form a molecular Ba hydride species, which undergoes alkene insertion and subsequent hydrogenolysis to close the catalytic cycle. Fe^0^ may play several roles: (i) the dissociative adsorption of H_2_ on the Fe^0^ surface can produce reactive hydrogen radicals that could convert Ba^0^ into soluble and highly reactive BaH_2_. (ii) Unsaturated substrates may be activated through adsorption as π-complexes on the Fe^0^ surface, thereby facilitating the hydride attack. (iii) Highly reactive H radicals formed on the Fe^0^ surface could directly react with alkylbarium intermediates to yield the reduced products. Consistent with the reaction mechanism for Ba^0^/Fe^0^, the combination of [(^Dipp^nacnac)MgH]_2_ and Fe^0^ also gives a highly active hydrogenation catalyst, which promotes the quantitative hydrogenation of benzene to cyclohexane. The proposed reaction sequence is illustrated in Fig. 7c. This mechanism involves the synergistic activation of benzene through coordination to the Fe^0^ surface and monomeric (^Dipp^nacnac)MgH.

In related work, Harder and colleagues showed that combining lithium aluminium hydride (LiAlH_4_) with finely divided metallic iron (Fe^0^) creates an effective catalyst for the stoichiometric and catalytic hydrogenation of unreactive alkenes and even arenes.^62^ While LiAlH_4_ or Fe^0^ alone are ineffective for these reactions, together they enable complete hydrogenation of internal and highly substituted alkenes, benzene, and even toluene at 50 bar H_2_ pressure and 120 °C. The elevated temperature and pressure are required for catalyst activation. After treatment at 120 °C and 50 bar H_2_, the catalyst remains active under mild conditions (*e.g.*, 1 bar H_2_ and ambient temperature for certain substrates). The proposed mechanism resembles those for Ba^0^/Fe^0^ and [(^Dipp^nacnac)MgH]_2_/Fe^0^. In the case of LiAlH_4_/Fe^0^, Fe^0^ catalyzes the decomposition of LiAlH_4_ into LiH and metallic Al, with Al^0^ reacting with H_2_ to form transient alane species (AlH_3_ or R_3_Al). Meanwhile, the Fe surface activates H_2_ and binds alkenes/arenes *via* π-interactions to facilitate insertion. This hypothesis is strongly supported by the high, induction-free activity of Al(^*i*^Bu)_3_/Fe.

Hevia and team reported the hydroamination of alkenes using a series of heterometallic alkali metal ferrates (Fig. 8).^88^ The trialkyl ferrate salts were obtained by co-complexation of (TMEDA)Fe(CH_2_SiMe_3_)_2_ with one equivalent of M(CH_2_SiMe_3_) (M = Li, Na, K, TMEDA = (*N*,*N*,*N*′,*N*′-tetramethylethane-1,2-diamine)) and Lewis donor TMEDA or PMDETA (*e.g.*, [Na(TMEDA)_2_][Fe(CH_2_SiMe_3_)_3_]) (Fig. 8a and b, PMDETA = *N*,*N*,*N*′,*N*″,*N*″-pentamethyldiethylenetriamine). The heterometallic ferrate complexes were anticipated to readily deprotonate the amine substrate due to an increased basicity, while also activating the alkene by the Lewis acidic alkali metal site. Consistent with this, the alkali metal ferrates efficiently catalyzed the intermolecular hydroamination of styrene with piperidine, showing a pronounced alkali metal cation effect [K^+^ (34%) ⟪ Li^+^ (89%) < Na^+^ (98%)] and markedly higher activity than the monometallic counterparts (TMEDA)Fe(CH_2_SiMe_3_)_2_ and Na(CH_2_SiMe_3_) (Fig. 8b). The reaction of [Na(TMEDA)_2_][Fe(CH_2_SiMe_3_)_3_] with piperidine afforded a tetranuclear complex containing six piperidide fragments, which proved equally effective in the hydroamination of styrene (Fig. 8c). Accordingly, alkali metal amido ferrates were proposed as catalytically active intermediates. Alkenes insert into the iron–amide bond to form an alkyl iron intermediate, which is protonated by amine to complete the catalytic cycle (Fig. 8c for M = Na).

**Fig. 8:**
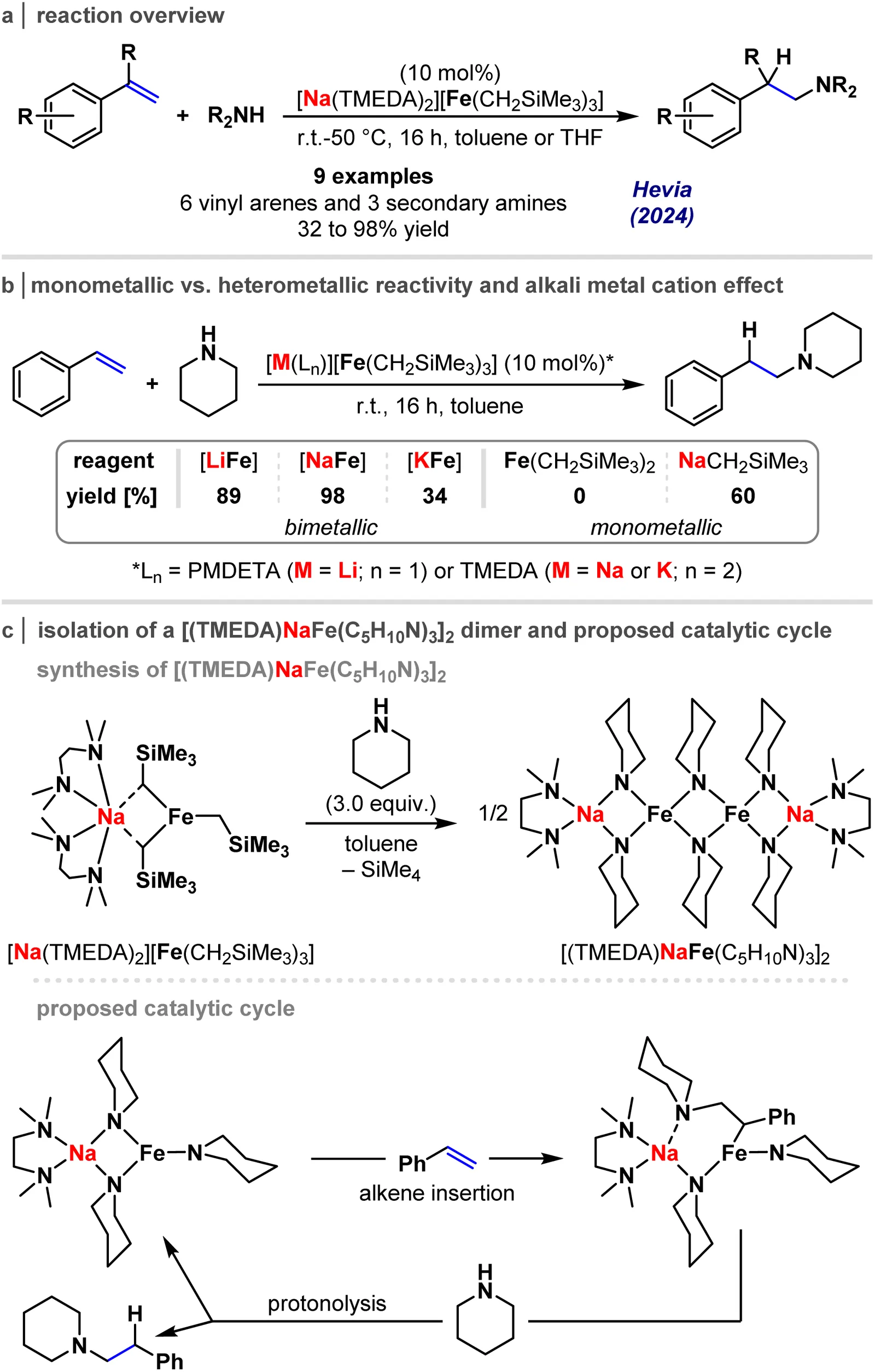
(a) Hydroamination of vinyl arenes catalyzed by [Na(TMEDA)_2_][Fe(CH_2_SiMe_3_)_3_]. (b) Comparison of homometallic *vs.* heterometallic reactivity and alkali metal cation effect. (c) Isolation of the catalytically active [(TMEDA)NaFe(C_5_H_10_N)_3_]_2_ and proposed catalytic cycle.

### 2.2 C–X (X = C, H, O, F, I) bond activation

Many important catalytic processes in organic synthesis rely on activating single bonds such as C–H, C–C, and C–O. Traditionally, this has been a domain of homometallic reagents.^89–93^ However, recent research emphasizes the role of heterometallic interactions in organometallic intermediates involved in these transformations. In particular, ionic 3d transition metalates have proven effective in activating various substrate classes through heterometallic mechanisms.

Hevia, Uzelac, and co-workers reported that alkali metal manganates M_2_Mn(CH_2_SiMe_3_)_4_ (M = Li, Na, K) promote the homocoupling of aryl iodides and terminal alkynes in the presence of an oxidizing agent (Fig. 9a and c).^94,95^ The heterometallic complexes can be generated either *in situ* by treating MCH_2_SiMe_3_ with MnCl_2_ or isolated as Lewis donor-stabilized solids. Various aryl iodides underwent homocoupling to afford a range of symmetrical bis(aryl) compounds in good to excellent yields.^94^ Investigations into the M–I exchange of 4-iodoanisole using different reagents showed almost quantitative exchange (95%) when using heterometallic Li_2_Mn(CH_2_SiMe_3_)_4_ (Fig. 9b). In contrast, Mn(CH_2_SiMe_3_)_2_ was completely inactive (0%), while Li(CH_2_SiMe_3_) gave a lower yield (62%). Additional evidence for the synergistic reactivity of Li and Mn was obtained from homocoupling reactions conducted in the presence of Lewis donors. Whereas TMEDA showed no effect, the lithium-specific sequestering agent 12-crown-4 completely inhibited the reaction, indicating that the close proximity of the lithium cation to the manganate anion is essential. Mechanistic studies further confirmed that intimate Li/Mn ion pairs are required to promote both Mn–I exchange and C(sp^2^)–C(sp^2^) bond formation. The isolation of a tetra-arylated lithium manganate complex showed that such species form biaryls upon exposure to O_2_ (Fig. 9c).

**Fig. 9:**
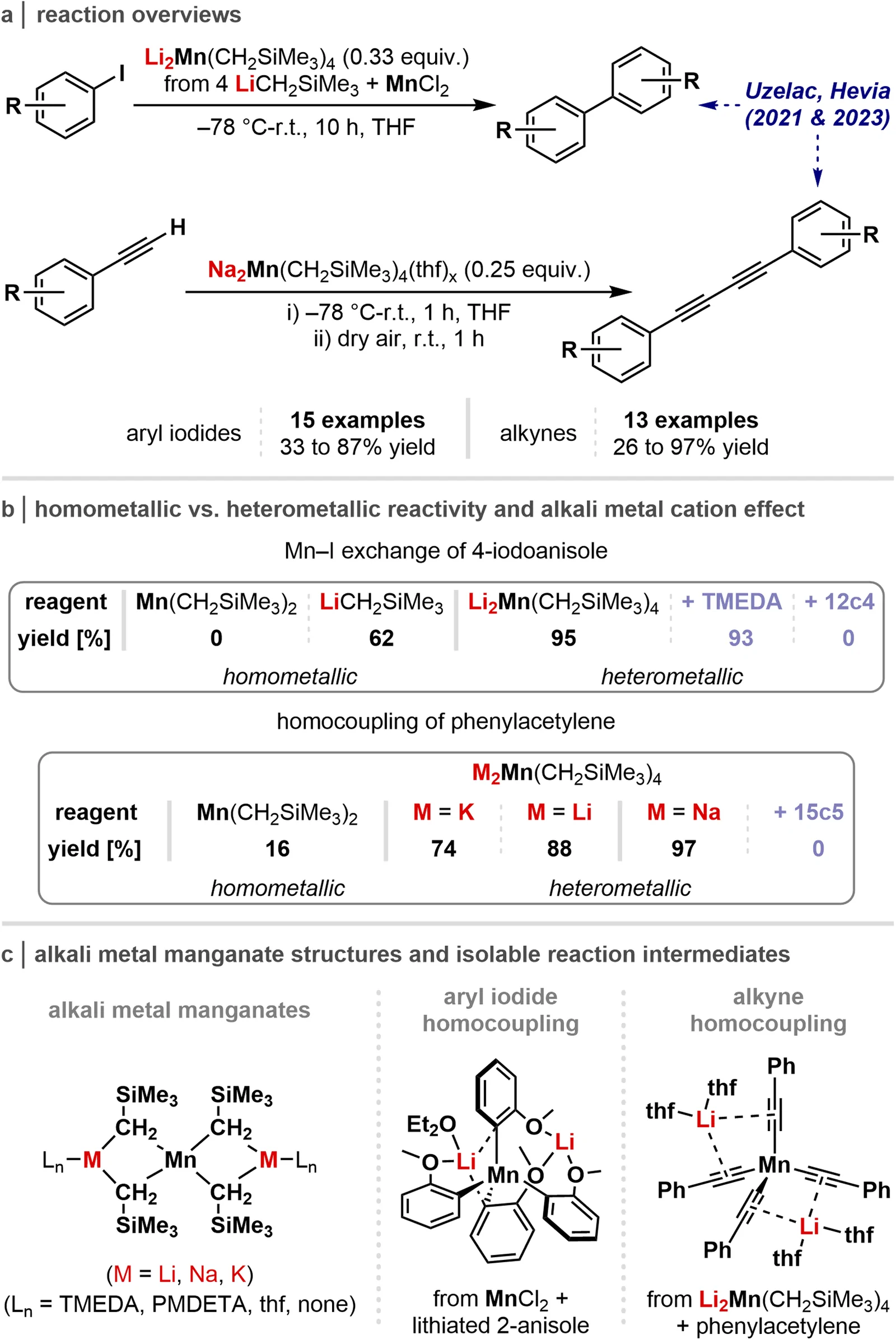
(a) Oxidative homocoupling of aryl iodides and alkynes mediated by alkali metal manganates. (b) Comparison of homometallic *vs.* heterometallic reactivity and alkali metal cation effect. (c) Structures of the tetra(alkyl) alkali metal manganates and aryl and alkyne manganate intermediates that undergo homocoupling upon oxidation.

Structurally analogous alkali metal manganates were employed in the oxidative homocoupling of terminal alkynes, affording a wide range of symmetric 1,3-diynes in good to excellent yields (Fig. 9a and c).^95^ The oxidative homocoupling of phenylacetylene demonstrated the direct influence of the alkali metal cation, following the trend: K^+^ (74%) < Li^+^ (88%) < Na^+^ (97%). Adding 15-crown-5 to the Na/Mn system completely inhibited the reaction (Fig. 9b). Structural analysis indicates that the process involves close alkali metal/Mn ion pairs, which can be prepared separately by treating terminal alkynes with M_2_Mn(CH_2_SiMe_3_)_4_ under inert conditions. An intermediate in the homocoupling of phenylacetylene was identified as a Li/Mn alkynyl complex that forms the 1,3-diyne upon exposure to dry air (Fig. 9c).

Further studies by Hevia, García-Melchor, and co-workers revealed the activation of C–H (and C–F)^96^ bonds in various fluoroaromatic substrates using a heterometallic Na/Fe complex (Fig. 10).^97–99^ The sodium ferrate was prepared by treating Fe(HMDS)_2_ (where HMDS is N(SiMe_3_)_2_) with NaHMDS to produce Na[Fe(HMDS)_3_]. This compound effectively metalates fluoroarenes at the C–H site next to a fluorine atom (Fig. 10a).^97^ The regioselective functionalization occurs only when the ate complex is present, whereas neither Na(HMDS) nor Fe(HMDS)_2_ alone activate the C–H bond.

**Fig. 10:**
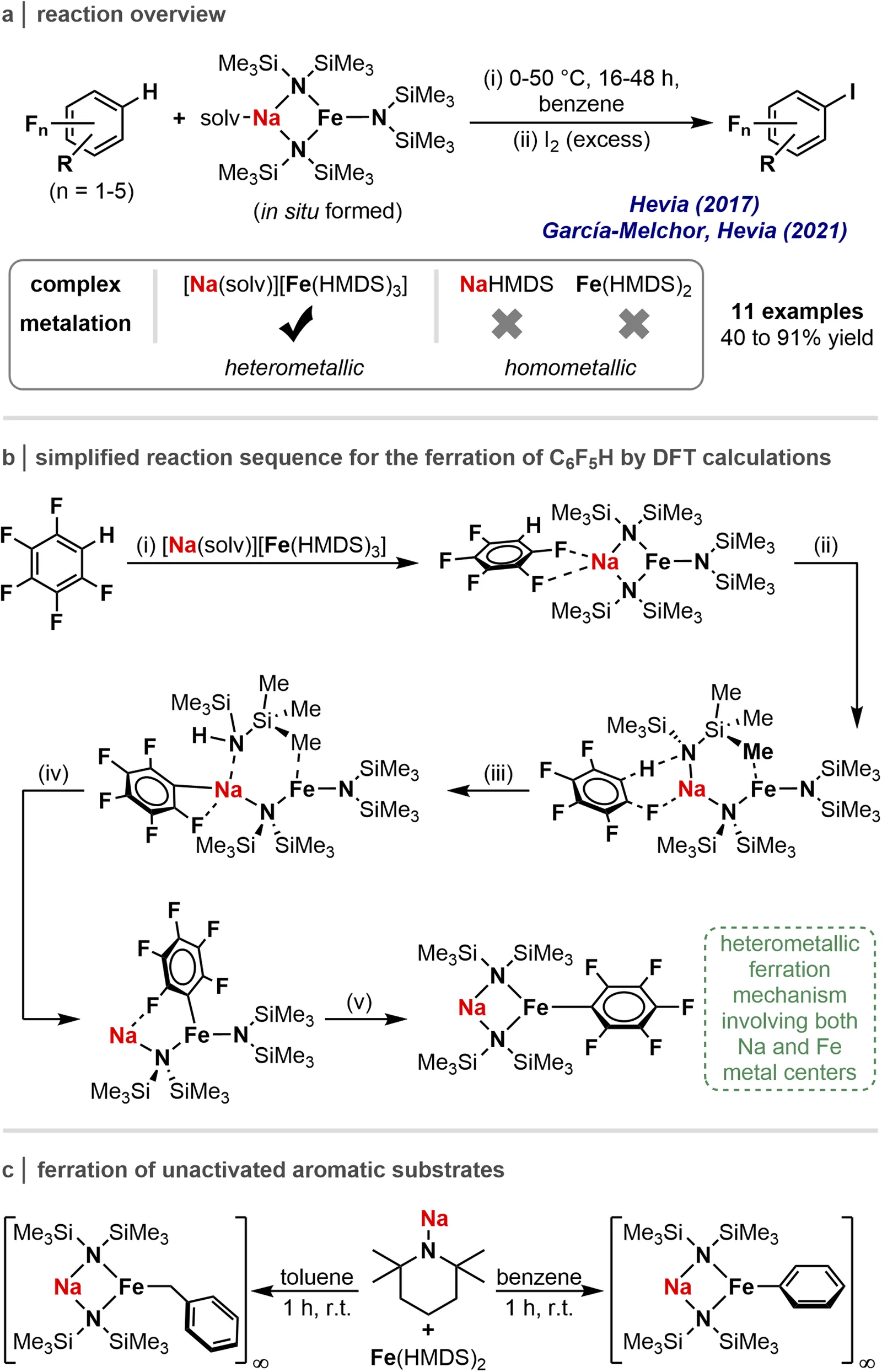
(a) Ferration of fluoroarenes mediated by [Na(solv)][Fe(HMDS)_3_]; solv = 1,4-dioxane. (b) Simplified reaction sequence for the ferration of C_6_F_5_H by DFT calculations. (c) Ferration of unactivated aromatic substrates.

Experimental and computational studies using C_6_F_5_H as model substrate have shown that the metalation is based on a stepwise sequence involving both metal centers in Na[Fe(HMDS)_3_] (Fig. 10b).^98^ The mechanism starts with C_6_F_5_H coordinating to Na *via* dative Na⋯F interactions (step i). Cleavage of one Fe–N_HMDS_ bond creates an intermediate where the amide is terminally bound to Na (step ii), allowing C_6_F_5_H to be metalated through a favorable transition state (step iii). This step can be described as an Na–H exchange, producing one equivalent of HN(SiMe_3_)_2_. Afterward, the fluoroaryl group undergoes intramolecular transmetalation by Fe in a barrierless process (step iv). The resulting heterometallic intermediate, with a covalent C–Fe bond and a dative Na⋯F bond, can be isolated as the dimer [Na(diox)_0.5_(C_6_F_5_)(HMDS)Fe(HMDS)]_2_ when the reaction occurs in the presence of 1,4-dioxane. In pure toluene, this intermediate isomerizes to a more thermodynamically stable complex where the fluoroaryl group is terminally bound to Fe (step v). These findings demonstrate the different roles of the metals: Na promotes the initial metalation, while Fe stabilizes the newly formed aryl anion. To further probe the role of the sodium amide in the metalation process, Na(HMDS) was substituted by the more basic and sterically hindered Na(TMP) (TMP = 2,2,6,6-tetramethyl-piperidinide, Fig. 10c). This modification facilitated the C–H activation of less reactive substrates such as toluene and benzene. This is attributed to a similar heterometallic mechanism involving the initial formation of Na[Fe(TMP)(HMDS)_2_] through co-complexation of the monometallic components. Because of the heteroleptic nature of this complex, ligand scrambling could produce various active species in solution, with Na[Fe(TMP)_3_] identified as the species having the lowest activation barrier.

Follow-up research by Hevia, García-Melchor and colleagues explored how sodium facilitates the cobaltation of fluoroarenes using a Na(HMDS)/Co(HMDS)_2_ system (Fig. 11a).^100–102^ Depending on the reaction conditions, either Na[Co(HMDS)_2_(Ar^F^)] (Ar^F^ = fluoroaryl), which shares the same structure as the ferration products discussed above, or the formation of homoleptic square planar [Na_2_Co(Ar^F^)_4_] complexes was observed.^100^ The latter tetra(aryl) cobalt complexes result from the rearrangement of Na[Co(HMDS)_2_(Ar^F^)], which also produces Na[Co(HMDS)_2_] and Co(HMDS)_2_. DFT studies on the reaction pathway leading to Na[Co(HMDS)_2_(Ar^F^)] indicate a stepwise metalation process driven by the interactions between both metal centers, similar to the mechanism suggested for Na(HMDS)/Fe(HMDS)_2_ (Fig. 10b). Depending on the fluoroarene substituents, the heterometallic mono(aryl) intermediates can be isolated or undergo ligand rearrangement to form the square planar complexes Na_2_[Co(Ar^F^)_4_]. Non-covalent interaction (NCI) and quantum theory of atoms in molecules (QTAIM) analyses of [Na_2_(solv)_*n*_Co(C_6_F_5_)_4_] identified stabilizing Na⋯F and Na⋯Co interactions that help maintain the square-planar structure through electrostatic and van der Waals forces.

**Fig. 11:**
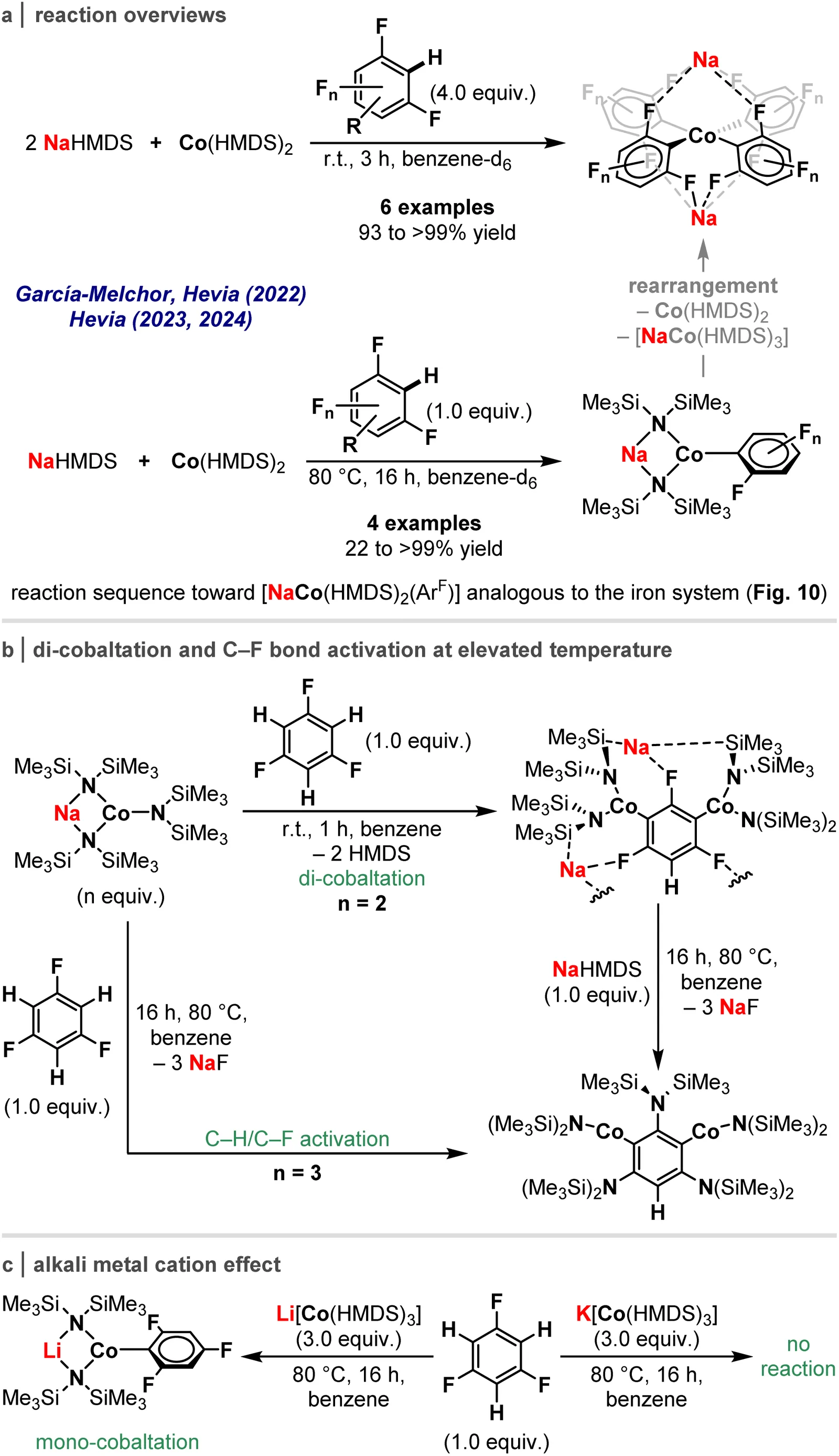
(a) Cobaltation of fluoroarenes mediated by Na(HMDS)/Co(HMDS)_2_. (b) Di-cobaltation and C–F bond activation at elevated temperature. (c) Alkali metal cation effect.

A subsequent study explored the multi-cobaltation and C–F bond activation of polyfluorinated arenes using Na[Co(HMDS)_3_] (Fig. 11b).^101^ The treatment of 1,3,5-trifluorobenzene with two equivalents of Na[Co(HMDS)_3_] at ambient temperature produced the di-cobaltation product [Na_2_Co_2_(C_6_F_3_H)(HMDS)_4_]. In contrast, three equivalents of Na[Co(HMDS)_3_] at 80 °C resulted in an unexpected substitution of fluoride by amide, leading to the dimetalated arene [1,3-(CoHMDS)_2_-2,4,6-(HMDS)_3_-C_6_H]. This process is believed to proceed *via* a cascade mechanism involving alternating eliminations of NaF and Co(HMDS)_2_, followed by additions of Co(HMDS)_2_ to benzyne intermediates. A significant alkali metal cation effect was observed, with no reaction between K[Co(HMDS)_3_] and 1,3,5-trifluorobenzene, while Li[Co(HMDS)_3_] only formed Li[Co(HMDS)_2_(C_6_F_3_H_2_)] *via* mono-cobaltation, regardless of stoichiometry or conditions employed (Fig. 11c).^102^

Hevia, Perrin, Payard, and co-workers conducted a series of investigations into the nickel-catalyzed cross-coupling of aryl ethers or fluoroarenes with organolithium and Grignard reagents (Fig. 12).^103–105^ Studies of the Ni(η^4^-cod)_2_-catalyzed cross-coupling reaction of 2-methoxynaphthalene and various [PhM(donor)_*n*_] aggregates (M = Li, MgCl; donor = none, THF, TMEDA) revealed significant effects of solvent and donor (Fig. 12a).^103^ In C_6_D_6_, the cross-coupling product was formed using donor-free PhLi, whereas in THF-d_8_, only *ortho*-lithiation of 2-methoxynaphthalene was observed. The stoichiometric coordination of PhLi with THF only slightly decreased the cross-coupling yield, but addition of bidentate TMEDA led exclusively to *ortho*-lithiation. These results indicate that lithium and nickel need to be closely positioned to achieve effective reactivity, with lithium coordinated by a labile donor such as THF that can be readily replaced.

**Fig. 12:**
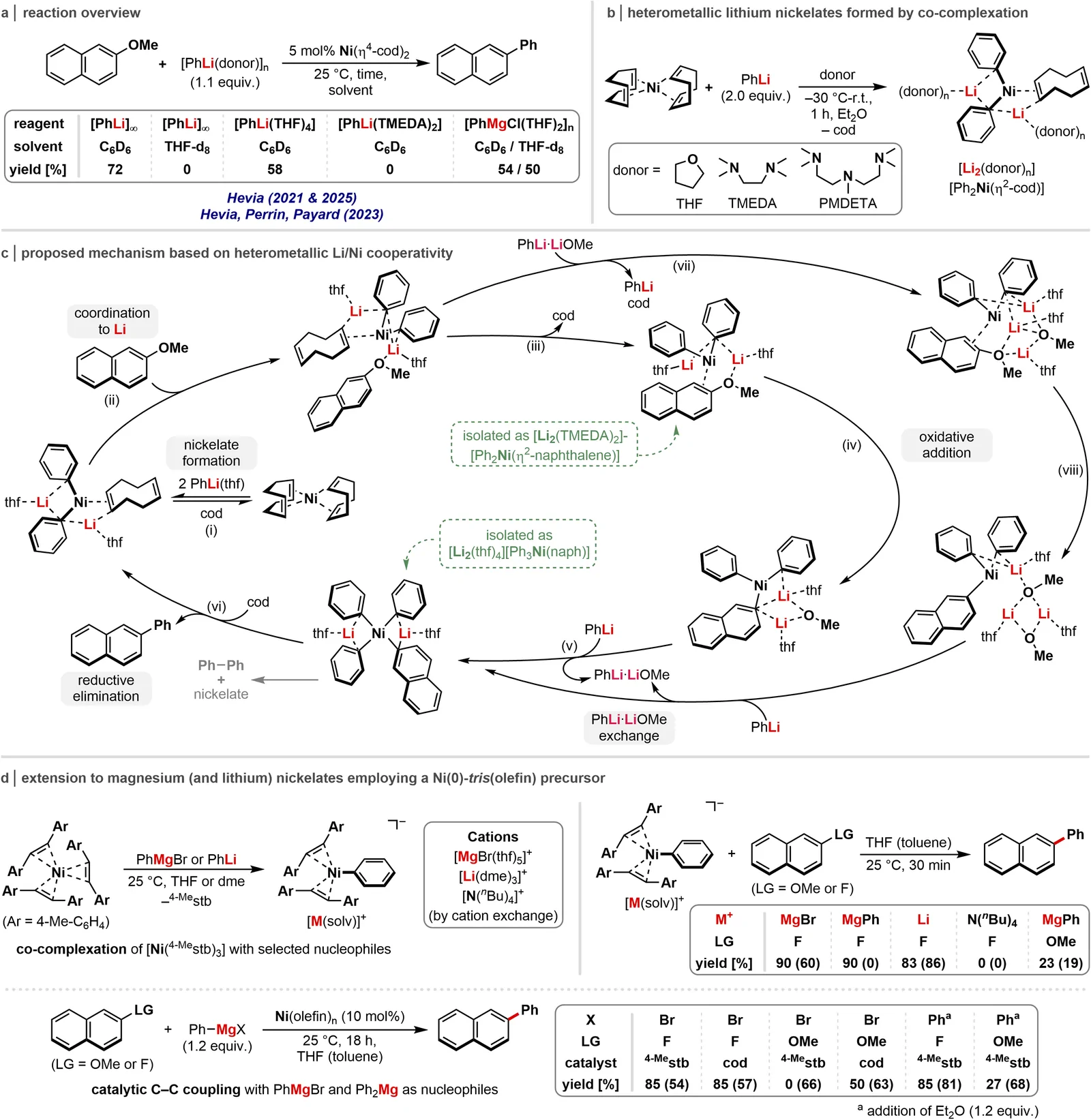
(a) Nickel-catalyzed C–C cross-coupling of aryl ethers with organolithium nucleophiles. (b) Formation of catalytically relevant lithium nickelates by co-complexation of Ni(η^4^-cod)_2_ with PhLi. (c) Proposed reaction mechanism for the C–C cross-coupling of 2-methoxynaphthalene with PhLi promoted by Li/Ni cooperativity. (d) Extension to magnesium (and lithium) nickelates employing [Ni(^4-Me^stb)_3_] (^4-Me^stb = (*E*)-4,4-dimethylstilbene) in the Ni-catalyzed C–C cross-coupling of aryl fluorides and aryl ethers with Grignard reagents. Yield in toluene is shown in brackets.

Reactions of Ni(η^4^-cod)_2_ with different amounts of PhLi produced well-defined lithium nickelates that form contact ion pairs in the solid state (Fig. 12b). The 1 : 2 Ni : PhLi species, [Li_2_(donor)_*n*_][Ph_2_Ni(η^2^-cod)] (donor = thf, TMEDA, PMDETA), were isolated and structurally characterized, showing close contacts between the Li^+^ cations and the phenyl and cod ligands at Ni. Replacing Ni(η^4^-cod)_2_ with nickelates in cross-coupling reactions resulted in nearly the same activity. For instance, using 5 mol% [Li_2_(thf)_4_][Ph_2_Ni(η^2^-cod)] and [PhLi(thf)_4_] gave the cross-coupled product in about 60% yield after similar reaction times, compared to 58% with Ni(η^4^-cod)_2_. Supported by additional structural, kinetic, and computational data, these findings indicate a reaction mechanism in which both the nucleophilic Ni center and the Lewis acidic Li^+^ cations act together (Fig. 12c).^104^

Starting from Ni(η^4^-cod)_2_, the addition of two equivalents of [PhLi(thf)] yields the 2 : 1 complex [Li_2_(thf)_2_][Ph_2_Ni(η^2^-cod)] while releasing one cod ligand (step i). The nickelate then binds 2-methoxynaphthalene *via* a Li⋯O interaction at the Lewis acidic Li^+^ cation (step ii). Next, π-coordination of the aryl ether substrate to Ni causes the cod ligand to release (step iii), followed by the rate-determining oxidative addition of the C–O bond at Ni, transferring the methoxy group to Li (step iv). The exchange of LiOMe with PhLi produces the tetraarylated lithium nickelate (step v), which then reductively eliminates the cross-coupling product and regenerates [Li_2_(thf)_2_][Ph_2_Ni(η^2^-cod)] (vi). The formation of LiOMe as a by-product may enable an alternative oxidative addition pathway with similar energy barriers, as seen in steps vii and viii. The isolation of the η^2^-arene and the tetraarylated lithium nickelates as donor complexes supports this mechanistic proposal. Subsequent research by Hevia and co-workers systematically explored the cross-coupling of Grignard (and organolithium) reagents using a Ni(0)-tris(olefin) complex (Fig. 12d).^105^ Co-complexation of Ni(^4-Me^stb)_3_ with organometallic salts produced solvent-separated ion pairs of the general structure [Cation][Ni(R)(^4-Me^stb)_2_] (Cation = MgX^+^, Li^+^ or N(^*n*^Bu)_4_^+^, R = aryl or alkyl, ^4-Me^stb = (*E*)-4,4-dimethylstilbene), including the phenyl nickelates featuring the anionic motif [Ni(C_6_H_5_)(^4-Me^stb)_2_]^–^. Reactivity studies using 2-fluoronaphthalene and 2-methoxynaphthalene as model substrates, along with different phenyl nickelates [Cation][Ni(C_6_H_5_)(^4-Me^stb)] as coupling reagents, afforded 2-phenylnaphthalene *via* challenging C–F and C–O bond activations. Although the nickelates have similar solid-state structures, their cross-coupling reactivity varies significantly depending on the countercation type and the solvent donor strength (THF *vs.* toluene). These findings imply that the C–X (X = F, OMe) bond cleavage involves similar heterometallic interactions between Ni and the s-block metal cation (MgX^+^ or Li^+^), which are most effective when the cation is in close contact with the organic substrate. Catalytic cross-couplings of 2-fluoronaphthalene and 2-methoxynaphthalene with PhMgBr and Ph_2_Mg as nucleophiles and Ni(^4-Me^stb)_3_ or Ni(η^4^-cod)_2_ as pre-catalysts (10 mol%) in different solvents further corroborated the central role of the s-block metal nickelates in these reactions.

Hevia, Stalke, and co-workers also reported the reduction of the 16 valence electron Ni(0) complex Ni(^4-Me^stb)_3_ to access a series of alkali metal nickelates [M_2_(thf)_*x*_][Ni(^4-Me^stb)_*n*_] (M = Li, Na, K, Rb, Cs; *x* = 1 or 2; *n* = 2 or 3) (Fig. 13a).^106^ The ionic complexes are formed either from direct reactions of Ni(^4-Me^stb)_3_ with alkali metals (M = Li, Na) and alkali metal graphites (M = K, Rb, Cs) or, for M = Li, by *in situ* reduction of Ni(η^4^-cod)_2_ and subsequent ligand exchange with ^4-Me^stb. The latter complex, [Li_2_(thf)_2_][Ni(^4-Me^stb)_2_], was studied by experimental charge density and QTAIM analyses, revealing a highly electron-rich nickelate (Bader charge at Ni: −0.63) with strong Ni → C=C_(olefin)_ π*-back-donation and polar Li⋯Ni interactions. Heterometallic complexes [M_2_(thf)_*x*_][Ni(^4-Me^stb)_*n*_] (M = Li, Na, K, Rb) successfully mediated the homocoupling of two equivalents of 2-fluoronaphthalene in THF (Fig. 13b). While all four complexes gave comparable yields for 2,2′-binaphthyl, a pronounced decrease in reaction rate from Li (1 h) to the heavier alkali metals (12 h for Rb) suggests that the cation is directly involved in the cross-coupling step. The stoichiometric reaction of 2-fluoronaphthalene with [Li_2_(thf)_2_][Ni(^4-Me^stb)_2_], followed by addition of 12-crown-4, afforded [Li(12c4)_2_][Ni(2-naphthyl)(^4-Me^stb)_2_] through C–F bond activation of the organic substrate (Fig. 12d). This complex was observed during the monitoring of the homocoupling of 2-fluoronaphthalene, and its direct reaction with 2-fluoronaphthalene produced 2,2′-binaphthyl in high yield, indicating its role in the homocoupling process. The same series of alkali metal nickelates was subsequently tested as catalysts in reductive alkene transformations (Fig. 13c). The hydrosilylation of norbornene and vinyl cyclohexane with SiH_2_Ph_2_, using 1 mol% pre-catalyst, gave over 95% yield within 10 minutes across all alkali metal nickelates. The exceptional turnover frequency of [Li_2_(thf)_2_][Ni(^4-Me^stb)_2_] (≥2500 h^−1^) highlights its effectiveness as a catalyst, on par with other nickel-based systems.^107,108^ Next, the reaction between norbornene and 1,4-cyclohexadiene was studied in the presence of 5 mol% alkali metal nickelate at 70 °C. No transfer hydrogenation occurred; instead, the norbornane dimer was selectively formed as the product of reductive alkene coupling. A clear alkali metal influence was observed, with product yields decreasing from Li (79%) to Na (52%) and then to K and Rb (53% and 25%). Additionally, vinyl cyclohexane was isomerized using 5 mol% nickelate pre-catalyst at 60 °C, yielding ethylidenecyclohexane.

**Fig. 13:**
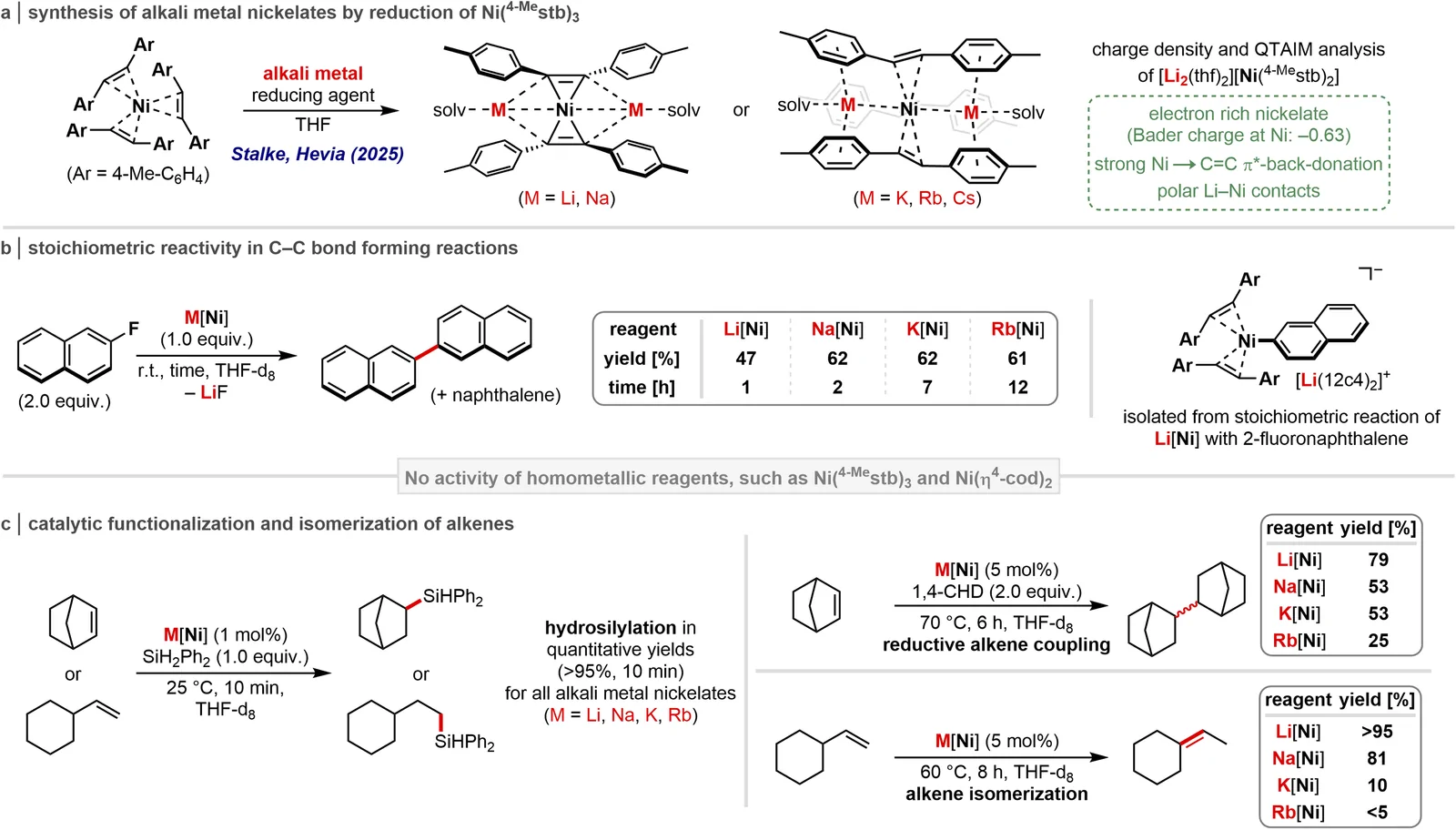
(a) Synthesis of highly reduced alkali metal nickelates by reduction of Ni(^4-Me^stb)_3_ (^4-Me^stb = (*E*)-4,4-dimethylstilbene). (b) Stoichiometric reactivity in C–C bond forming reactions. (c) Catalytic functionalization and isomerization of alkenes.

The isomerization yields varied significantly depending on the alkali metal cation, following the trend: Li^+^ (>95%) > Na^+^ (81%) > K^+^ (10%) > Rb^+^ (<5%). The reaction may proceed *via* a radical pathway starting with substrate coordination to Ni. This pathway is preferred in the two-coordinate Li and Na nickelates but is hindered in the three-coordinate K and Rb nickelates. Notably, neither stoichiometric nor catalytic reactivity can be reproduced using homometallic Ni(0) precursors such as Ni(^4-Me^stb)_3_ and Ni(η^4^-cod)_2_.

In this context, Hevia, Barcells and team also investigated the C–C bond oxidative addition of biphenylene to alkali metal nickelates (Fig. 14).^109^ The complexes of the type [M_2_(solv)_*n*_]-[Ph_2_Ni(η^4^-biphenylene)] (M = Li, Na, K; solv = dme, tetrahydropyran [thp], PMDETA) were prepared by reaction of Ni(*ttt*-CDT) (where *ttt*-CDT is *trans*,*trans*,*trans*-1,5,9-cyclododecatriene) with two equivalents of PhLi and one equivalent of biphenylene (for M = Li), or through alkali metal exchange using MO^*t*^Bu (M = Na, K) starting from the lithium nickelate generated *in situ*. In these η^4^-biphenylene complexes, the cleavage of the C–C bond to form the ring-opened analogs strongly depends on the alkali metal cation and its coordination environment (Fig. 14a). With the Li^+^ complex, biphenylene underwent oxidative addition to Ni in just 15 minutes upon warming from −30 °C to ambient temperature, whereas the Na and K analogs required 3 and 12 hours, respectively.

**Fig. 14:**
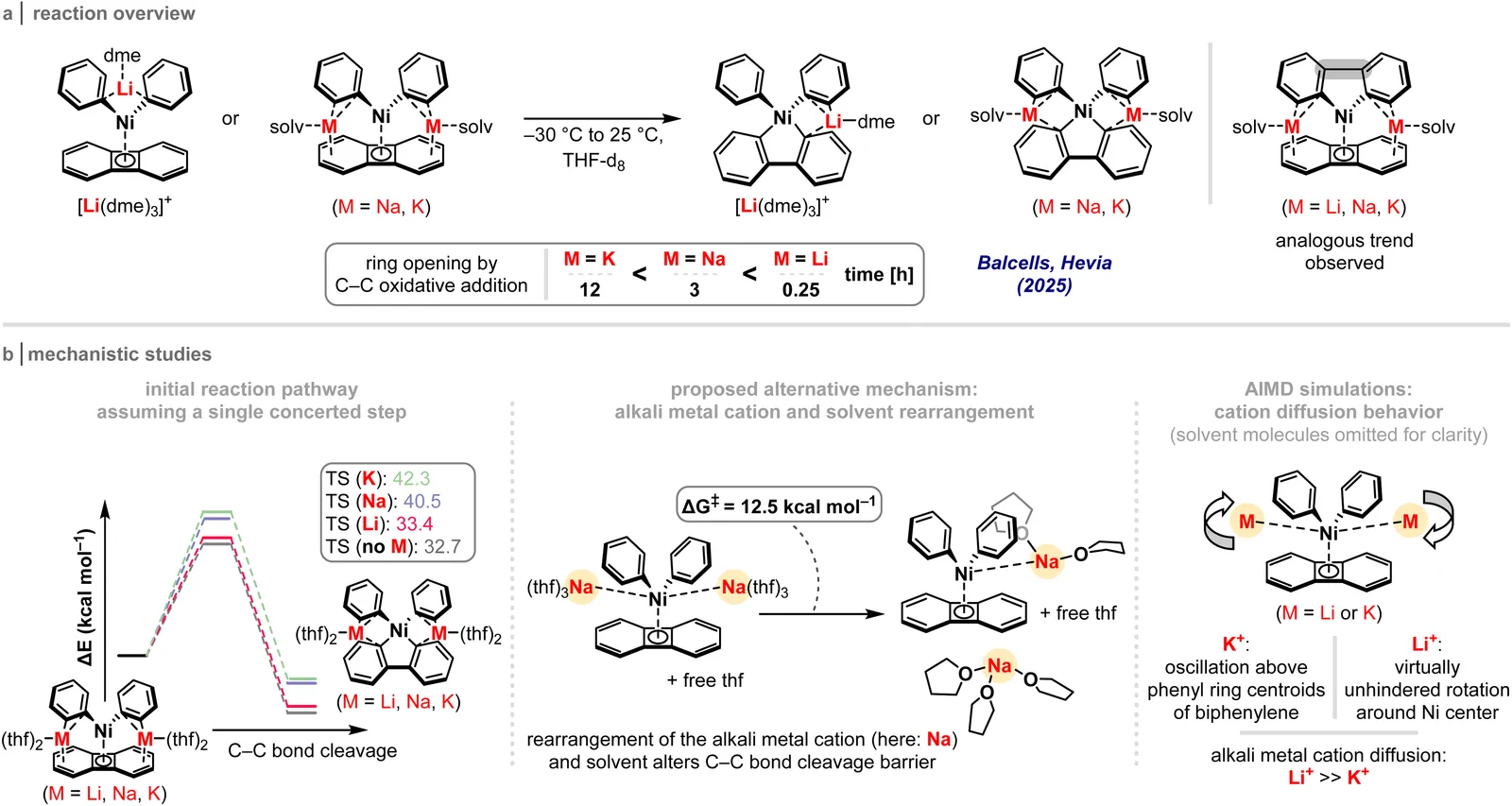
(a) Ring opening of biphenylene that is η^4^-coordinated to alkali metal nickelates (M = Li, Na, K). (b) Mechanistic studies including DFT calculations on the reaction pathway and AIMD simulations analyzing the alkali metal cation diffusion behavior.

DFT modeling of the C–C bond cleavage as a single concerted step successfully replicated the experimentally observed reactivity trend, with Li^+^ being the most reactive (Li^+^ ⟫ Na^+^ 〉 K^+^), although the calculated energy barriers (for instance, 33.4 kcal mol^−1^ for Li) were higher than those suggested by the experimental rates (Fig. 14b, left). NMR kinetic studies of the ring opening of [Na_2_(thp)_4_][Ph_2_Ni(η^4^-biphenylene)] determined an experimental activation barrier of 18.6 kcal mol^−1^. Additional computational analyses of the sodium nickelate ion pair demonstrated that the location of the alkali metal cation and its solvation are decisive in determining the magnitude of the C–C bond cleavage barrier (Fig. 14b, middle). The migration of one sodium cation to the opposite side of the biphenylene plane and concurrent solvent reorientation (12.5 kcal mol^−1^) generates an intermediate that undergoes C–C bond cleavage with a reduced barrier of 10.6 kcal mol^−1^, consistent with the experimental activation barrier for the sodium nickelate. *Ab initio* molecular dynamics (AIMD) simulations of the η^4^-biphenylene alkali metal nickelates (M = Li and K) revealed that the solvation dynamics are strongly dependent on the identity of the alkali metal cation (Fig. 14b, right). In a 10.3 ps simulation at −33 °C, the Li^+^ cations rotate around the Ni center with minimal restrictions, whereas the K^+^ cations oscillate in largely fixed positions above the phenylene rings. This behavior is also reflected in the radial distribution of the alkali metal cations relative to the phenyl ring centroids (of biphenylene), which is narrow and overlapping for K^+^ but considerably broader and more stochastic for Li^+^. The virtually unrestricted movement of Li^+^ likely facilitates the adoption of a favorable geometry en route to C–C bond cleavage.

An emerging area of research concerns the chemistry of heterometallic beryllium compounds. This area has recently been reviewed, so only some of the most salient results are summarized here.^110^ A handful of structures featuring discrete Be–M bonds have been isolated to date (Fig. 15a), which contain a TM atom in addition to Be. An early example was the complex [(Cy_3_P)_2_Pt–Be(Cl)X] (X = Cl, CH_3_) by Braunschweig and co-workers, which shows an electron-precise Be–Pt bond.^111^ More recently, the synthesis and isolation of diberyllocene, CpBeBeCp, by Aldridge, Boronski, and co-workers has facilitated the preparation of other compounds with Be–M bonds, such as [CpBeZn(^Dipp^nacnac)] and [Ni(BeCp)_6_].^112,113^*C*_3v_ symmetric [Ni(BeCp)_6_] was prepared by reaction of [Ni(η^4^-cod)_2_] with CpBeBeCp (three equivalents). Quantum chemical studies indicated that the complex exhibits an inverted ligand field and that seven-center two-electron bonding between Ni and Be imparts aromatic character. Beyond the synthesis of structurally unusual complexes, the introduction of beryllium-based ligands induces novel reactivity to transition metal complexes. Aldridge, Boronski, and co-workers reported that the photochemical beryllation of methane (and benzene) by CpBeBeCp is catalyzed using CpMn(CO)_3_ and Cp*Re(CO)_3_ (Cp* = C_5_Me_5_) (Fig. 15b).^114^ The isolation of heterometallic intermediates, such as [*trans*-CpMn(BeCp)_2_(CO)_2_], and their use as pre-catalysts indicate synergistic reactivity between both metals during the C–H beryllation process. Quantum chemical calculations of the CH_4_ activation mechanism identified a key transition state in which Mn and two Be atoms cooperatively mediate the C–H bond cleavage. The high Lewis acidity and σ-donating character of the Be centers generate an electron-rich Mn center, and together these features are crucial for the observed reactivity. In contrast, calculations for the analogous CH_4_ borylation with [*trans*-CpMn(Bpin)_2_(CO)_2_] indicate that the lower Lewis acidity of the boryl ligands is insufficient to promote C–H activation. In agreement with this, no equivalent transformation has been documented for the boryl analogue.

**Fig. 15:**
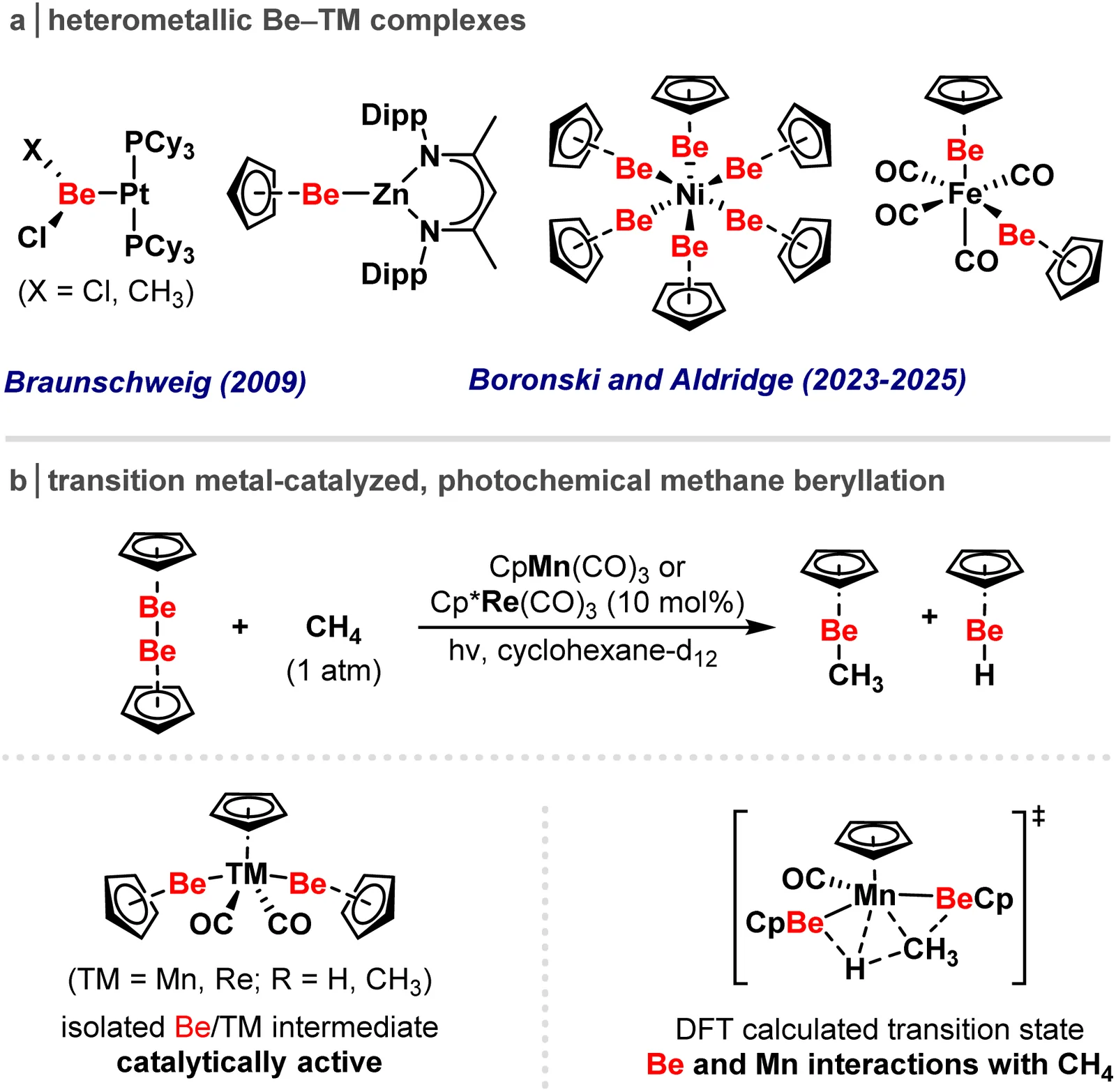
(a) Heterometallic Be–TM complexes. (b) Photochemical beryllation of methane catalyzed by transition metal complexes.

### 2.3 Isomerization and related reactions

Most of the examples discussed in Sections 2.1 and 2.2 involve d-block metal anions that form tight ion pairs with s-block metal cations. An alternative approach integrates s-block metal cations *via* coordination through supramolecular ligand frameworks such as crown ethers.^115–117^ While cation-responsive systems of this type have been reported to catalyze various reactions, including hydroformylation,^118^ hydrogenation,^119^ and C–H oxidation,^120^ analysis of the underlying cation effects have remained limited.

Miller and co-workers developed an iridium hydride [(κ^5-15c5^NCOP^^*i*^Pr^)Ir(H)]^+^ featuring a macrocyclic aza-15-crown-5 pincer ligand for cation-controlled dihydrogen activation (Fig. 16a and b).^121^ The ligand can reversibly switch between different coordination modes when binding to a substrate. This process can be modulated by the addition of an alkali metal cation, with the specific effect depending on the cation's type and concentration. Hydrogen–deuterium exchange with this complex under a D_2_ atmosphere was slow without an alkali metal cation, indicating that the weakly coordinating D_2_ substrate does not bind effectively to the metal center (Fig. 16a). In contrast, the addition of substoichiometric MBAr^F^_4_ (M = Na, Li; Ar^F^ = 3,5-bis(trifluoromethyl)phenyl) resulted in a 20-fold rate increase for Na^+^ and an approximately 250-fold rate enhancement for Li^+^. The reaction rate was also greatly influenced by the alkali metal cation concentration, rising significantly as the loading was raised from substoichiometric to superstoichiometric amounts (0.3 equiv. Na^+^: *t*_1/2_ = 8 h *vs.* 1.2 equiv. Na^+^: *t*_1/2_ = 2 h). The alkali metal cation is proposed to promote D_2_ coordination by binding to the aza-15-crown-5 macrocycle and displacing the crown ether oxygen cis to the hydride ligand (Fig. 16a and c). The hemilabile ligand adopts a tridentate coordination mode, which is suggested to accelerate hydrogen activation and scrambling. The most significant increase is seen with Li^+^, which binds more selectively to the crown ether motif than Na^+^.

**Fig. 16:**
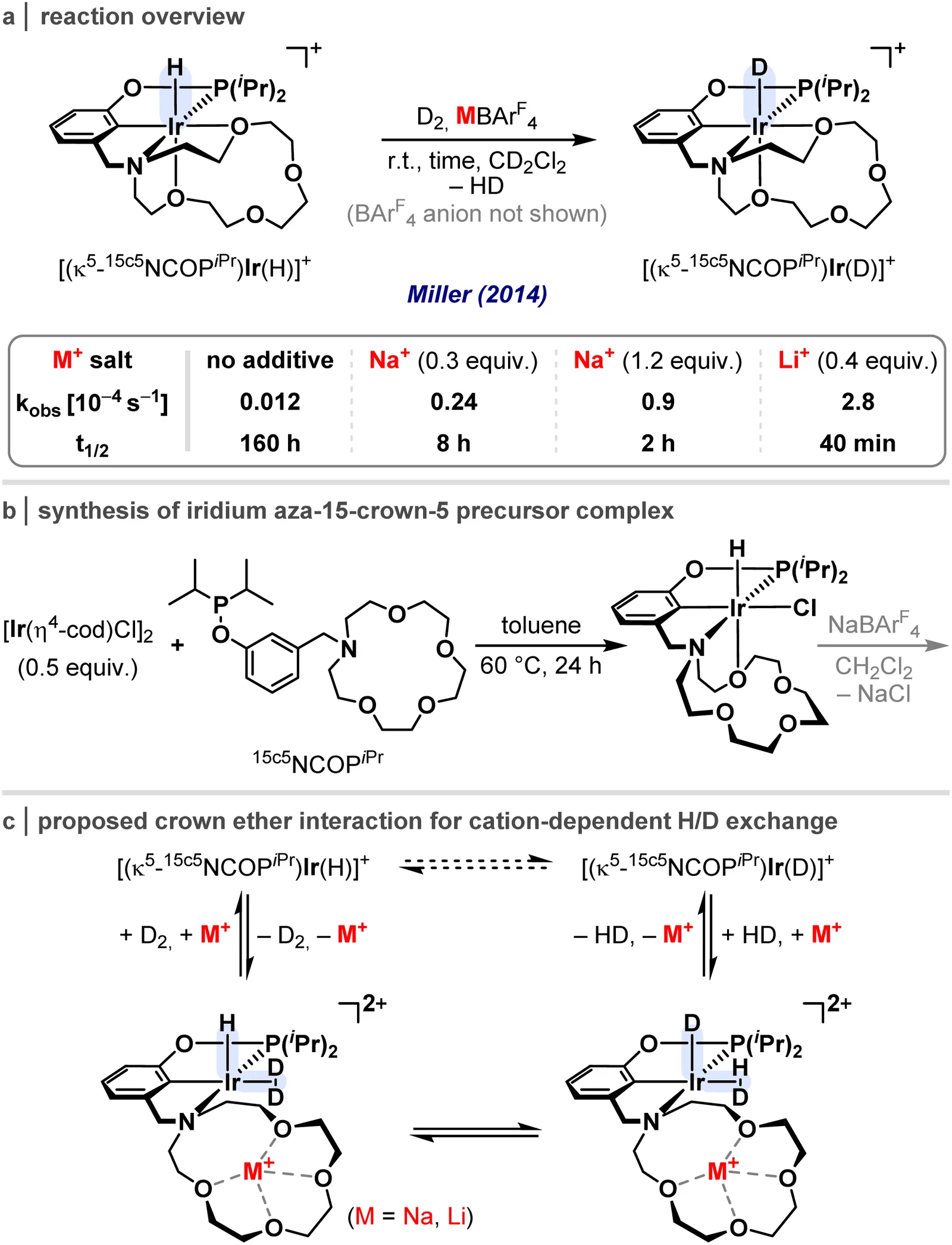
(a) H/D exchange with an iridium pincer-crown ether complex and MBAr^F^_4_ (M = Na, Li; Ar^F^ = 3,5-bis(trifluoromethyl)phenyl). (b) Synthesis of the iridium aza-15-crown-5 precursor complex. (c) Proposed crown ether interaction for cation-dependent H/D exchange.

Subsequently, the Miller team reported an ion-responsive catalytic alkene isomerization using the same complex (Fig. 17).^122,123^ This is an ideal test reaction for counterion effects because it requires only the direct coordination of a single substrate to the transition metal center, minimizing the potential for side reactions (Fig. 17). In the absence of alkali metal cations, [(κ^5-15c5^NCOP^^*i*^Pr^)Ir(H)]^+^ (1 mol%) catalyzed the slow conversion of allylbenzene to β-methylstyrene over 141 h, with an initial turnover frequency (TOF) of 1.8 h^−1^ (Fig. 17a).^115^ The modest activity of the iridium complex is attributed to the hemilabile crown ether ligand *cis* to the hydride, which can be displaced by the alkene substrate, albeit with an equilibrium that disfavors alkene binding. The addition of MBAr^F^_4_ (M = Li, Na, K) to the iridium catalyst significantly accelerated the isomerization to β-methylstyrene, with a rate enhancement of up to 1000-fold for Li^+^ (TOF = 2000 h^−1^). Based on kinetic and spectroscopic analyses, the significant increase in rate is associated with a change in the reaction mechanism, which involves a parallel catalytic cycle facilitated by the iridium complex bound to the alkali metal cation (Fig. 17b). Here, the hemilabile crown ether macrocycle is fully dissociated from the transition metal center through cation–crown interactions, giving rise to a κ^3^-coordinated pincer ligand instead of the κ^4^-coordination present without an alkali metal cation. This results in a change in the reaction order in allylbenzene from first to zero, indicating a shift in the resting state and turnover-limiting step from crown ether displacement by alkene substrate to the insertion/elimination process at iridium.

**Fig. 17:**
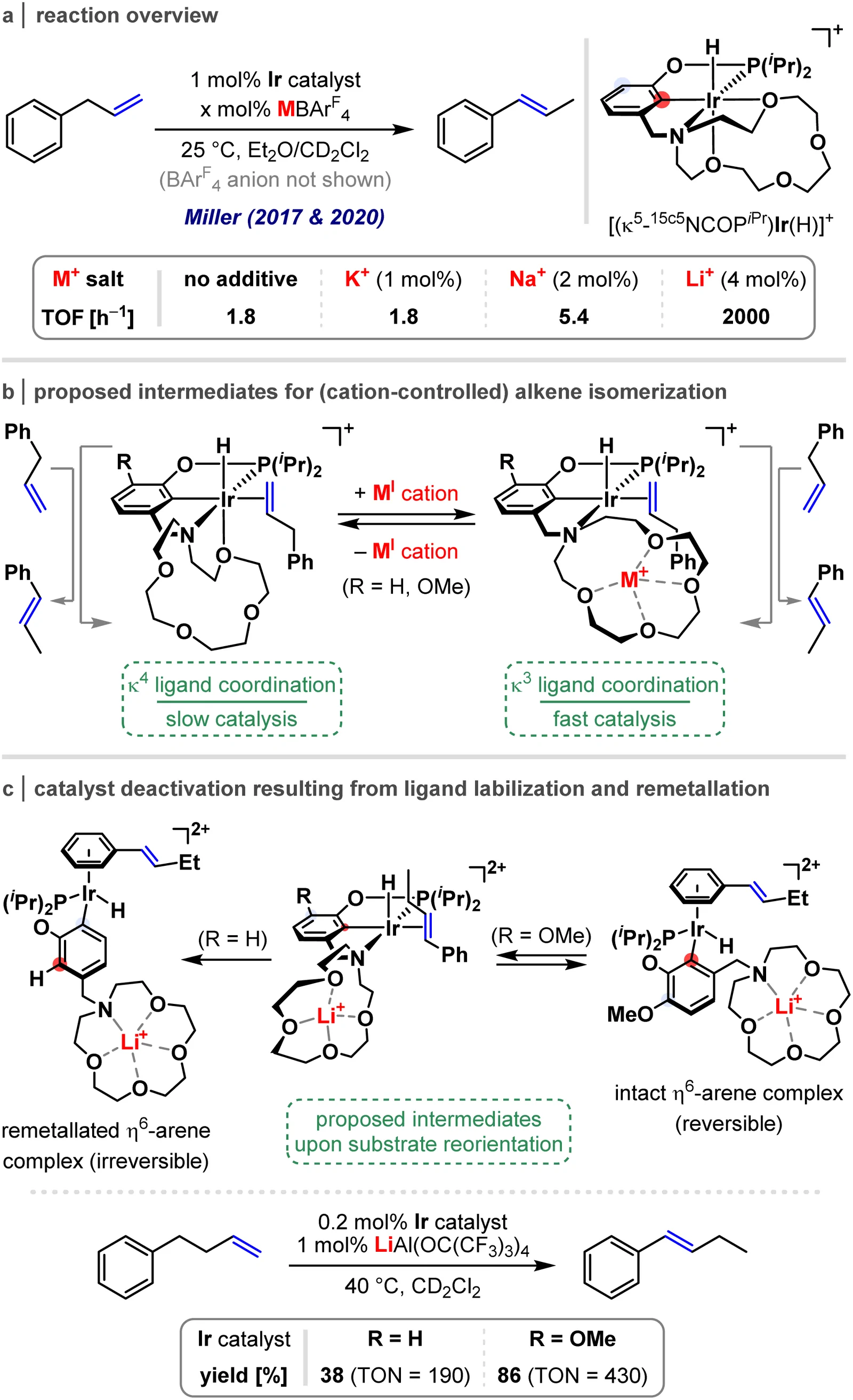
(a) Cation-controlled isomerization of allylbenzene with an iridium pincer-crown ether complex. (b) Proposed catalyst structures and alkali metal cation interaction for the isomerization reactions. (c) Catalyst deactivation by ligand labilization and remetallation.

Further mechanistic studies with [(κ^5-15c5^NCOP^^*i*^Pr^)Ir(H)]^+^ identified a catalyst deactivation pathway initiated by amine dissociation, which leads to metallation at a different position on the aryl backbone of the pincer and formation of a much less active η^6^-arene complex (Fig. 17c).^123^ Although the remetallation process was irreversible in the parent catalyst system (R = H), adding a methoxy group to the arene backbone produced an enhanced catalyst that stayed active under the same reaction conditions. Use of the modified complex [(κ^5^-MeO-^15c5^NCOP^^*i*^Pr^)Ir(H)]^+^ in the multipositional isomerization of 4-phenyl-1-butene resulted in significantly higher activity than the original catalyst [(κ^5-15c5^NCOP^^*i*^Pr^)Ir(H)]^+^ (TON = 430 *vs.* 190).

In a further study, Miller and co-workers designed a cation-controlled pincer-crown-ether iridium catalyst that converts terminal alkenes to two distinct internal isomers (Fig. 18).^124^ The complex features an extended aza-18-crown-6 ether motif, which binds alkali metal cations through pentadentate coordination. The catalytic isomerization of 1-butenes bearing different functional groups revealed that the 18-crown-6-based catalyst promotes multipositional isomerizations, with regio- and stereoselectivity dependent on the presence of an alkali metal cation. Without a salt additive, the isomerization of 4-phenyl-1-butene selectively produced the 2-alkene (93%, *E* : *Z* = 14 : 1) (Fig. 18a). In contrast, the addition of NaBAr^F^_4_ shifted the selectivity to the 3-alkene (94%, *E* : *Z* = 50 : 1).

**Fig. 18:**
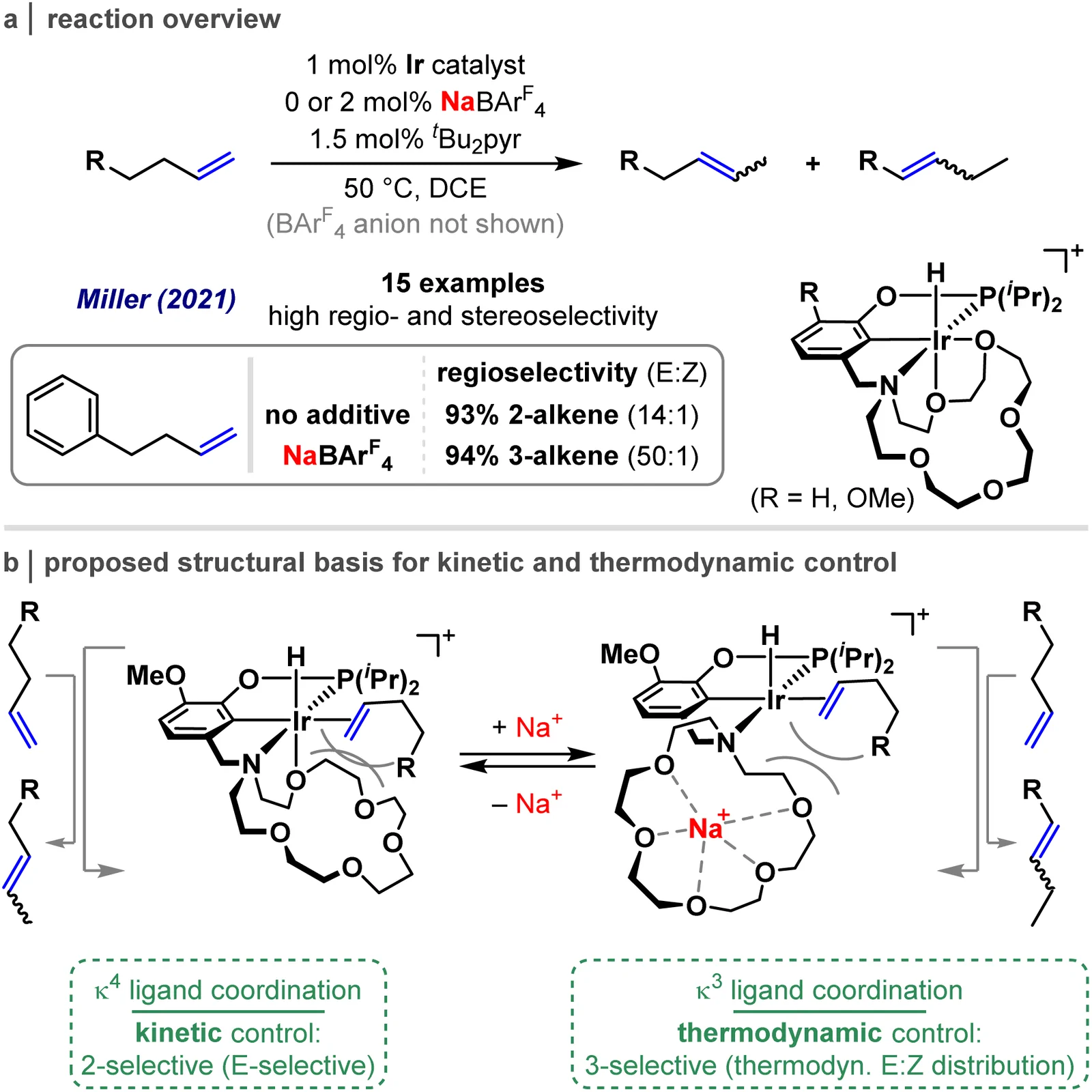
(a) Positional isomerization of alkenes using a cation-responsive iridium pincer-crown ether complex. (b) Proposed catalyst structures and alkali metal cation interaction underlying kinetic and thermodynamic reaction control.

The mechanism for the positional isomerization depends on noncovalent interactions that direct the catalyst toward either kinetic or thermodynamic control (Fig. 18b). Without NaBAr^F^_4_, the 2-alkene forms as a kinetic product through a catalyst that adopts a tetradentate (κ^4^) binding mode at Ir, with one ether oxygen attached to Ir during turnover. Steric constraints probably allow the 2-alkene formation but hinder further isomerization to the 3-alkene due to kinetic barriers. When NaBAr^F^_4_ is present, the 3-alkene regioisomer is formed selectively under thermodynamic control, when the 3-alkene is more stable than the other isomers. This isomerization occurs through a catalyst with a tridentate (κ^3^) binding mode at Ir, where strong Na^+^-crown interactions break the coordination of ether oxygens to Ir. The resulting less crowded coordination environment likely reduces barriers to isomerization, favoring the formation of the most stable regioisomer under thermodynamic control.

Later, the Miller group explored a series of iridium pincer catalysts for alkene isomerization and hydrogenation to better understand how cation-induced rate enhancement occurs (Fig. 19).^125^ They designed three catalysts with varying binding affinities for alkali metal cations to determine whether cation-tunable ligand hemilability or electric field effects caused by the cations^126,127^ govern the catalytic activity (Fig. 19a and b). The former mechanism involves blocking the alkene substrates from reaching the active iridium site, while the latter suggests that the isomerization rate is boosted by local electric fields from nearby alkali metal cations. This study revealed that the macrocycle-containing catalyst ^15c5^Ir exhibits the expected switchable behavior. In the presence of 2.2 mol% LiBAr^F^_4_·3OEt_2_, ^15c5^Ir (2 mol%) is highly active toward the isomerization of 1-triisopropyl-silyloxy-but-3-ene into the 2-alkene (130-fold rate acceleration) (Fig. 19a). In contrast, the bis(methoxyethyl)-based catalyst ^BME^Ir displayed generally low activity and only a modest response to Li^+^, with a 2.7-fold increase in the rate of 2-alkene formation. This behavior is attributed to insufficient donor interactions of the methoxyethyl substituents and Li^+^, preventing meaningful modulation of the substrate-binding equilibrium. The diethylamine-based catalyst ^Et^Ir, containing a labile DME ligand, was highly active in the first transposition of the alkene substrate, both in the absence and presence of Li^+^, indicating that switchable catalysis is precluded because the alkene substrate is not effectively hindered from binding to the Ir site. A mechanism based on electric field effects would predict low activity for ^BME^Ir and ^Et^Ir; however, substrate gating instead implies that ^Et^Ir should operate rapidly with or without Li^+^ due to the labile dme ligand (Fig. 19b). These results strongly imply that substrate gating is the dominant mechanism.

**Fig. 19:**
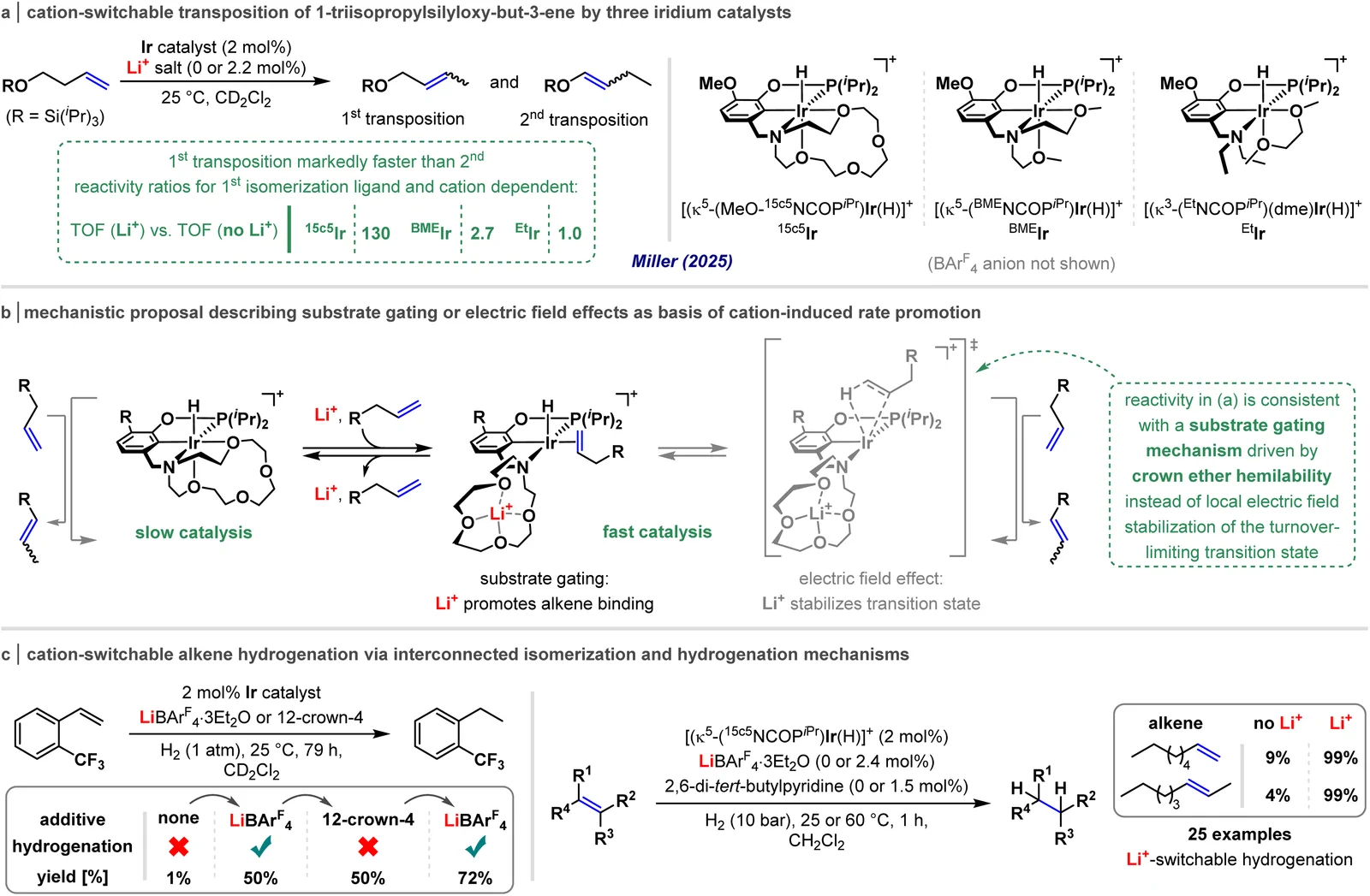
(a) Cation-switchable transposition of 1-triisopropylsilyloxy-but-3-ene by three iridium pincer catalysts with and without Li^+^ salt additive. (b) Mechanistic proposal describing substrate gating or electric field effects as basis of cation-induced rate promotion. (c) Cation-switchable alkene hydrogenation *via* interconnected isomerization and hydrogenation mechanisms.

Investigations of alkene hydrogenation using the cation-responsive catalyst ^15c5^Ir established the generality of substrate gating as a strategy to control reactions of alkenes (Fig. 19c). Hydrogenation of *o*-(trifluoromethyl)styrene with 2 mol% ^15c5^Ir occurs in the presence of catalyst-bound Li^+^ but not in its absence, demonstrating switchable catalysis. Mild reaction conditions were sufficient to hydrogenate cyclic, mono-substituted, and 1,2-disubstituted alkenes, whereas more highly substituted and long-chain alkenes required heating to 60 °C. For some substrates, isomerization and hydrogenation proceed at similar rates, generating a new alkene that is rapidly hydrogenated at the isomerized position.

### 2.4 Polymerization reactions

The combination of an s-block element with a 3d transition metal has become an increasingly explored strategy to improve catalyst activity and selectivity in polymerization catalysis. This trend is reflected in the growing number of heterometallic systems designed to harness the synergistic reactivity of two distinct metal centers in ring-opening polymerization (ROP) and copolymerization (ROCOP).^128–130^

Williams and co-workers developed a highly active heterodinuclear Co(ii)Mg(ii) complex for the copolymerization of CO_2_ with epoxides (Fig. 20).^131^ The mixed-metal catalyst features a macrocyclic tetraaza-bis(olate) ligand, which accommodates both metals in a planar coordination environment (Fig. 20a). When employed in the reaction of cyclohexene oxide (CHO) under 1 bar of CO_2_ pressure and in the presence of 1,2-cyclohexane diol (1,2-CHD) as chain transfer agent, the Co(ii)Mg(ii) catalyst displayed markedly superior catalytic activity compared to its homodinuclear Co(ii)Co(ii) and Mg(ii)Mg(ii) analogues (TOF = 1205 h^−1^*vs.* 712 h^−1^ and 368 h^−1^, respectively). The polymerization is proposed to follow a chain shuttling mechanism, which is based on the alternate binding of the growing polymer chain to the two metal centers through either carbonate or alkoxide coordination (Fig. 20b). The rate-determining step involves coordination of the epoxide at Mg(ii), which is attacked by the carbonate ligated to Co(ii).

**Fig. 20:**
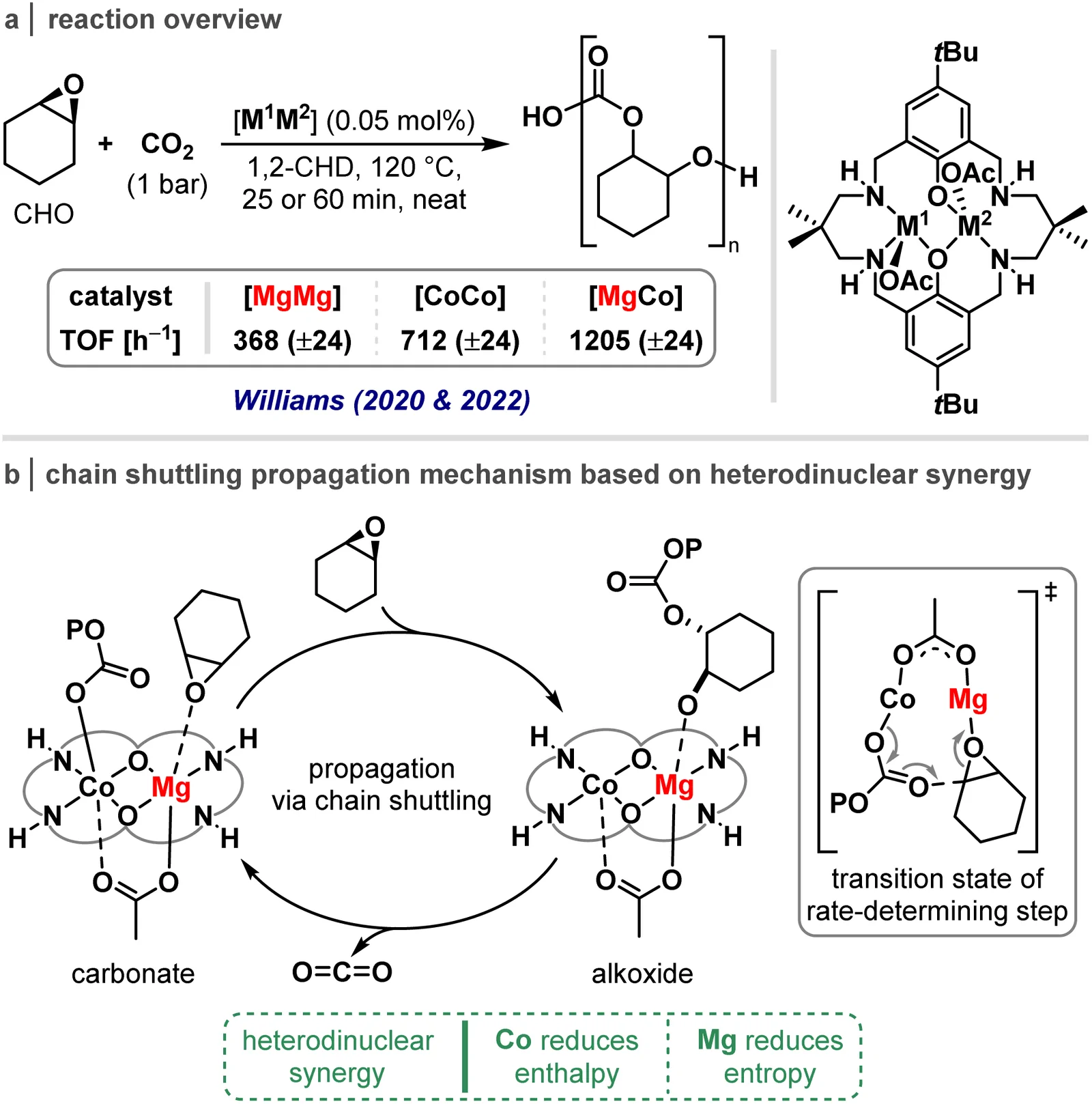
(a) Ring-opening copolymerization of cyclohexene oxide (CHO) and CO_2_ catalyzed by a heterodinuclear Co(ii)Mg(ii) catalyst (1,2-CHD = 1,2-cyclohexane diol). (b) Chain shuttling propagation mechanism based on heterodinuclear synergy.

Kinetic analyses indicate that each metal independently contributes to reducing the overall kinetic barrier. Co(ii) decreases the transition state enthalpy by increasing the nucleophilicity of the metal carbonate, while Mg(ii) reduces the activation entropy associated with epoxide coordination due to its low bond directionality and higher oxophilicity. Together, these complementary effects render the heterodinuclear complex the most active catalyst. The synergistic reactivity was preserved across a series of other Mg(ii)TM(ii) catalysts, including TM = Mn, Fe, and Ni, all of which exhibited superior ROCOP activity compared to the homodinuclear Mg(ii)Mg(ii) catalyst.^132^

Following the successful application of the heterodinuclear Mg(ii)TM(ii) catalysts, the Williams group explored the pairing of group 1 metals (M = Na, K, Rb, Cs) with a salen-based Co(iii) complex for ROCOP catalysis (Fig. 21).^133,134^ The dinuclear complexes consist of a Co(iii) atom positioned within the Schiff base cavity, adjacent to a macrocyclic crown ether moiety that binds the group 1 metal through dative O⋯M^+^ interactions (Fig. 21a). Evaluation of the dinuclear catalysts in the ROCOP of propylene oxide (PO) and CO_2_ (20 bar) in the presence of 1,2-CHD revealed a pronounced influence of the alkali metal cation, following the general reactivity trend K(i) ⟫ Na(i) ⟫ Rb(i) 〉 Cs(i) in turnover frequency. TOF values of up to 800 h^−1^ were reached using the Co(iii)K(i) catalyst under optimized conditions and at low loadings. Based on kinetic analyses, the rate law is first order in both catalyst and epoxide, suggesting a dinuclear chain shuttling mechanism involving analogous carbonate and alkoxide intermediates to those proposed for the Co(ii)Mg(ii) system (Fig. 21b).

**Fig. 21:**
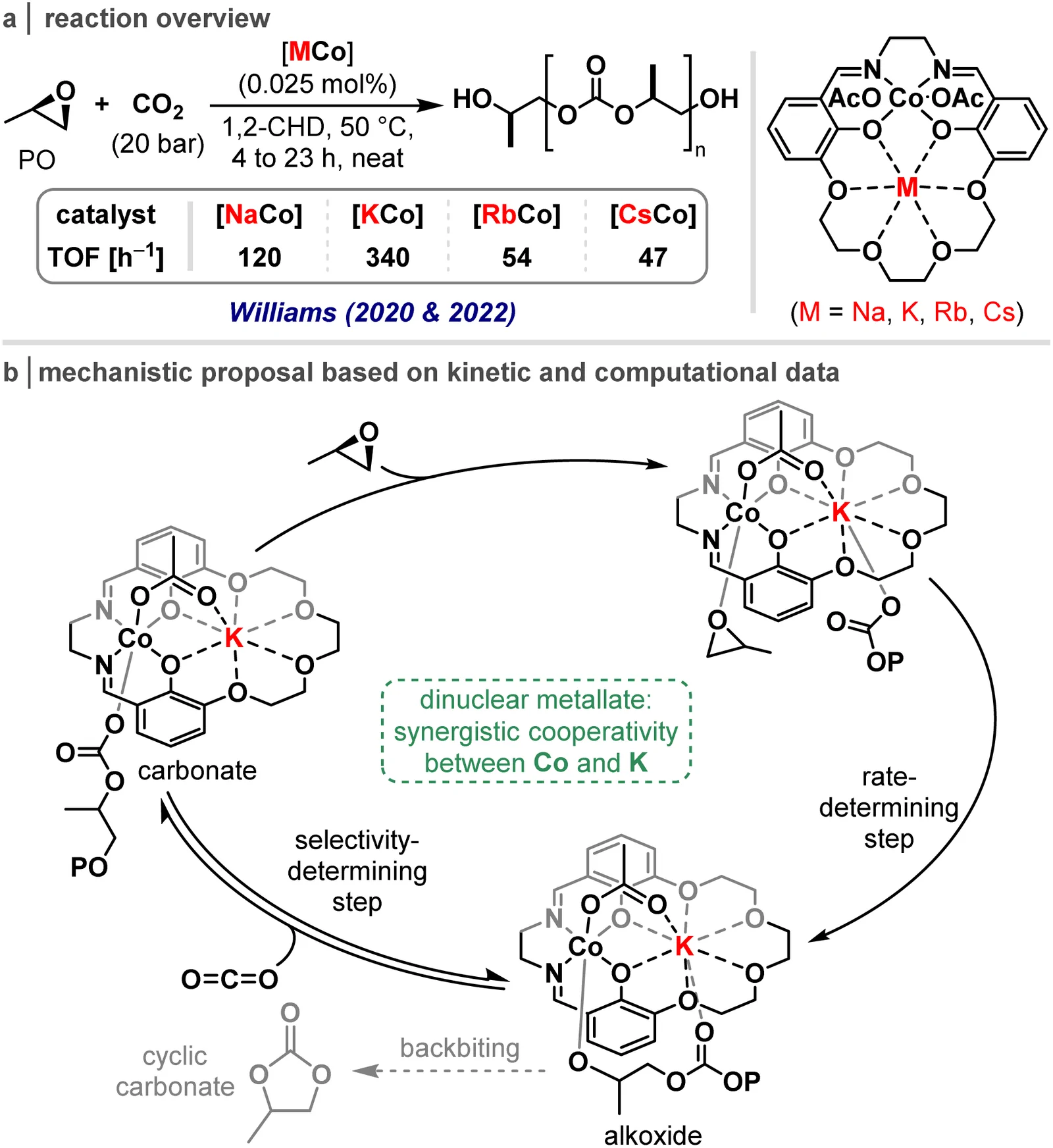
(a) Ring-opening copolymerization of propene oxide (PO) and CO_2_ catalyzed by heterodinuclear Co(iii)M(i) catalysts (M = Na, K, Rb, Cs). (b) Proposed chain shuttling mechanism.

Detailed DFT studies of the copolymerization catalyzed by Co(iii)K(i) examined two potential propagation pathways to understand the role of the two different metals and identify the rate-determining step. Modelling of the propylene oxide coordination at either the Co(iii) or K(i) center revealed rate-determining barriers for the PO ring opening of approximately 22 kcal mol^−1^ and 36 kcal mol^−1^, respectively. Under the typical reaction conditions (50 °C, 20 bar CO_2_), the calculated barrier suggests that the potassium-activated pathway is unlikely to occur experimentally. In contrast, the calculated lower barrier for the cobalt-activated mechanism compares well with the experimentally determined value at 50 °C. Accordingly, both alkoxide and carbonate intermediates are suggested to coordinate to the cobalt center during chain propagation, with the potassium center providing transient stabilization of the polymer carbonate group in the rate-determining PO ring-opening step (Fig. 21b). Additional polymerization selectivity analyses examining the formation of propylene carbonate *via* backbiting of the alkoxide intermediate revealed an energy barrier comparable to that of the epoxide ring-opening step. Experimental studies identified a CO_2_-dependent insertion equilibrium between the alkoxide and carbonate intermediates, where decreasing CO_2_ pressure shifts the equilibrium toward the alkoxide species and enhances backbiting.

Subsequent investigations of Co(iii)M(i/ii) catalysts (M(i/ii) = Na(i), K(i), Ca(ii), Sr(ii), and Ba(ii)) across several polymerization reactions, including PO/CO_2_ ROCOP, PO/phthalic anhydride (PA) ROCOP, CHO/CO_2_ ROCOP, and CHO/PA ROCOP, yielded quantitative relationships between catalytic activity, selectivity and s-block metal Lewis acidity (Fig. 22a).^135,136^ The acidity was described using literature-reported p*K*_a_ values of the s-block metal aqua complexes.^137^ Notably, no correlations were observed between catalytic performance and either the ionic radius of the s-block metal cation or its binding strength to the crown ether motif. Maximum activities (and selectivities) for all investigated reactions were achieved with the Co(iii)K(i) catalyst, consistent with the trend of decreasing Lewis acidity across M(i/ii). Depending on the reaction conditions and the nature of the key reaction intermediate, catalyst activity exhibited either exponential or linear correlations with the p*K*_a_ values of the s-block metal aqua complexes (Fig. 22b). In the CHO/CO_2_ ROCOP, an exponential correlation in catalyst activity was observed at a CO_2_ pressure of 20 bar, which was attributed to the s-block metal carbonate serving as key intermediate under these conditions. Here, decreasing the s-block metal Lewis acidity is proposed to destabilize metal-carbonate species and lower the activation barrier for epoxide ring-opening. In contrast, a linear correlation was observed when decreasing the CO_2_ pressure to 1 bar, which was attributed to a metal-alkoxide species serving as the key reaction intermediate. Analogous to the observations in PO/CO_2_ ROCOP (Fig. 21b), a shift in the insertion equilibrium is likely responsible for the change in the activity-Lewis acidity relationship. At low CO_2_ pressures, the linear increase in activity with decreasing Lewis acidity reflects a shift of the insertion equilibrium toward the alkoxide intermediate, whereas at high pressures, the equilibrium favors the carbonate intermediate. A similar structure-activity correlation was recently reported for CHO/PA ROCOP catalyzed by complexes incorporating Fe(iii) with different s-block metals, M(i/ii) = Na(i), K(i), Sr(ii), Ba(ii).^138^ The p*K*_a_ values of the s-block metal aqua complexes were once more used as a measure for the Lewis acidity. The less Lewis acidic M(i) catalysts (M = Na and K) outperform the more Lewis acidic M(ii) catalysts (M = Sr and Ba), supporting the hypothesis that nucleophilic activation occurs at the s-block metal while activation of the electrophile occurs at the iron atom. A kinetic study using microscale calorimetry provided a detailed understanding of the synergistic behavior of the two metal atoms, revealing how different catalyst compositions affect the enthalpy (Δ*H*^‡^) and entropy of activation (Δ*S*^‡^). The results show that, while no simple correlation exists between Lewis acidity (p*K*_a_) of the s-block metal cation and Δ*H*^‡^ or Δ*S*^‡^ individually, the p*K*_a_ values serve as a quantitative metric for predicting catalytic activity as determined by the combined effect of Δ*H*^‡^ and Δ*S*^‡^ (*i.e.*, Δ*G*^‡^ = Δ*H*^‡^ − *T*ΔS^‡^).

**Fig. 22:**
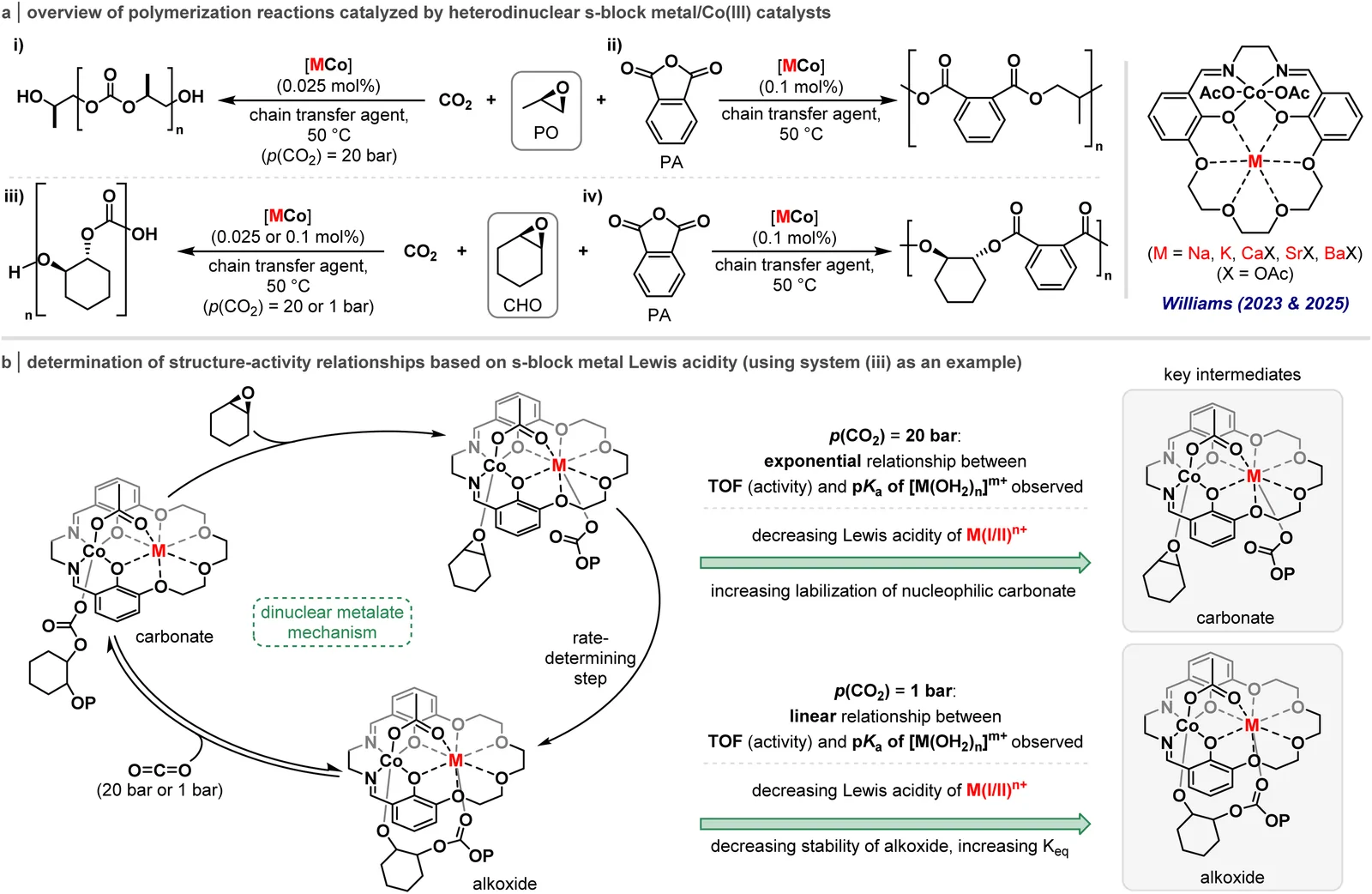
(a) Overview of polymerization reactions catalyzed by heterodinuclear s-block metal/Co(iii) catalysts: (i) PO/CO_2_ ROCOP; (ii) PO/PA ROCOP; (iii) CHO/CO_2_ ROCOP; (iv) CHO/PA ROCOP. (b) Determination of structure–activity relationships based on s-block metal Lewis acidity.

Similar effects have been observed in alkene polymerization employing heterometallic complexes that combine s- and d-block metals. In this approach, the transition metal drives polymerization, while the s-block metal modulates alkene coordination and insertion steps through stereoelectronic tuning. Do and co-workers investigated the polymerization of ethylene using a series of nickel complexes that are supported by dinucleating phenoxyimine ligands bearing a pendant polyethylene glycol (PEG) side chain (Fig. 23).^139–141^ Upon addition of MBAr^F^_4_ salts (M = Li, Na, K, Cs), heterometallic complexes were formed with both 1 : 1 and 1 : 2 alkali metal-to-nickel ratios in solution, depending on the length of the ether chain (Fig. 23a; first-generation catalysts). Introducing a methylene spacer at the beginning of the PEG chain (*n* = 4) favored the formation of alkali metal–nickel complexes in a 1 : 1 ratio, which was expected to improve polymerization activity (Fig. 23a; second-generation catalysts).

**Fig. 23:**
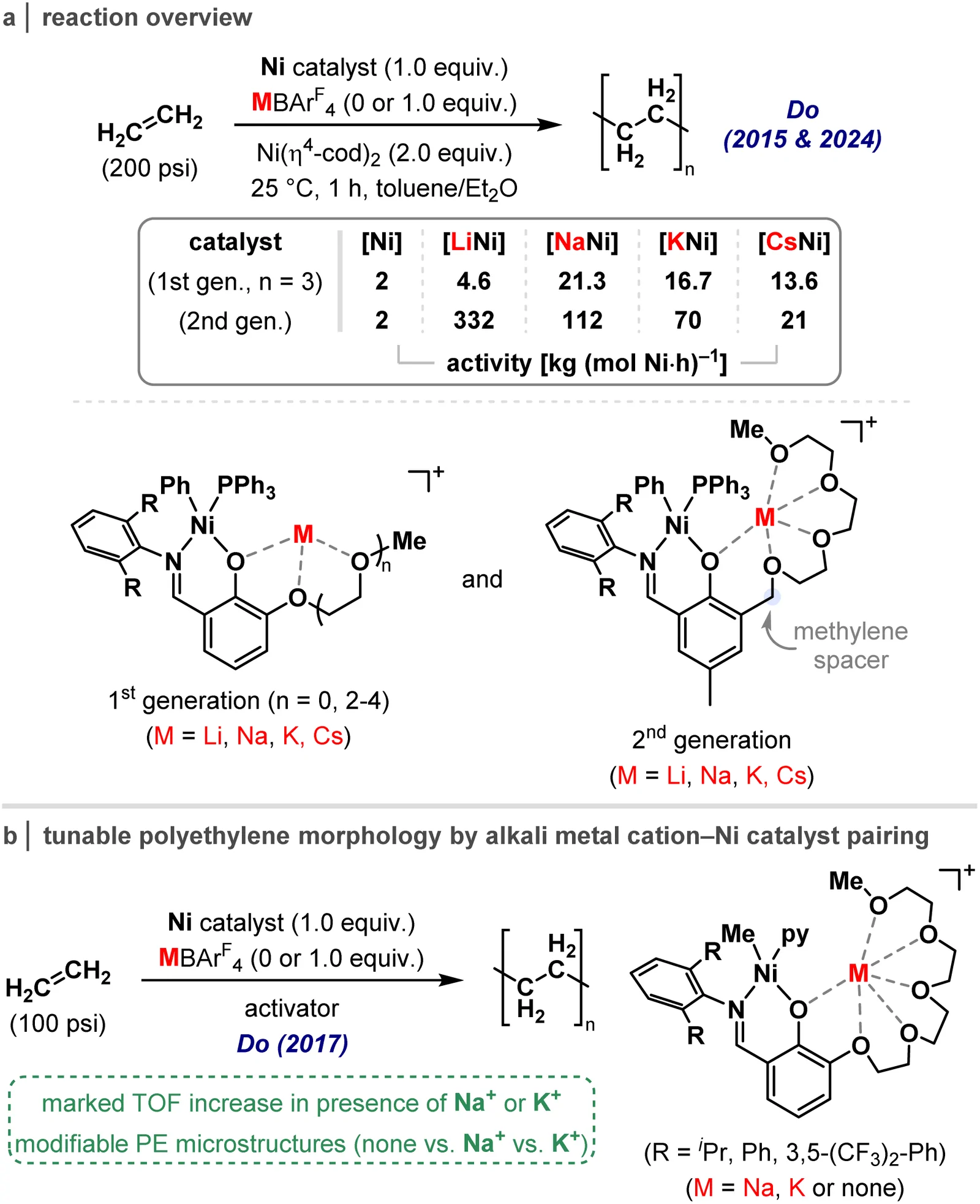
(a) Polymerization of ethylene using alkali metal cation-responsive nickel phenoxyimine catalysts. (b) Tunable polyethylene morphology by alkali metal cation–Ni catalyst pairing.

Two key observations were made when testing both catalyst types in the polymerization of ethylene (200 psi), alongside Ni(η^4^-cod)_2_ as a phosphine scavenger: the first-generation alkali metal–nickel catalysts with matching PEG side chains showed improved performance relative to their mononuclear analogues, a behavior that correlated with the high association constants of the resulting heterometallic complexes. Addition of NaBAr^F^_4_ led to a significant increase in activity for ligands with long PEG chains (*n* = 3 or 4), while the shortest-chain ligand (*n* = 0) showed reduced activity when the metal salts were introduced. The increase in activity with stronger cation binding is attributed to the alkali metal enhancing the electrophilicity of the Ni center, thereby facilitating ethylene coordination and insertion.

Furthermore, the second-generation catalysts exhibited a clear trend in polymerization activity (Li^+^ > Na^+^ > K^+^ > Cs^+^) and were generally more active than the previously reported catalysts. The observed behavior is attributed to the selective formation of 1 : 1 alkali metal–nickel complexes, with the modified site positioning the alkali metal cation closer to the nickel center. This is proposed to increase the electrostatic charge and electron induction at Ni. When using modified catalysts with an extended PEG chain (*n* = 5), the choice of alkali metal and aryl imine substituents affected the microstructure and molecular weight of polyethene formed, as well as the polymerization rate (Fig. 23b). The heterodinuclear complexes displayed significantly higher activity than the mononuclear Ni catalysts, with Na(i)Ni(ii) achieving the highest TOF values within the series.

Later, Do and team reported on ethylene polymerization using structurally related alkali metal–nickel catalysts containing a phenoxyphosphine-PEG ligand (Fig. 24).^142,143^ The crystallographic characterization of a series of 1 : 1 complexes (M = Li^+^, Na^+^, K^+^, and Cs^+^) enabled quantification of their steric bulk through buried volume analysis (Fig. 24a). The alkali metal cations provide substantial steric shielding in the heterometallic catalysts relative to a conventional monometallic reference. In addition, cyclic voltammetry measurements of the mono- and heterometallic complexes revealed a positive shift in the oxidation peak following the trend: Li^+^ > Na^+^ > K^+^ > Cs^+^, relative to the mononuclear analogue. The incorporation of alkali metal cations resulted in a shift of the anodic peak consistent with their relative Lewis acid strength, suggesting that these cations increase the effective charge at the nickel center. The polymerization of ethylene (450 psi) at 30 °C catalyzed by the *in situ* generated alkali metal–nickel complexes reproduced the activity trend Li^+^ > Na^+^ > K^+^ > Cs^+^, consistent with the preceding structural findings. Notably, the heterometallic catalysts were significantly more active than the corresponding mononuclear nickel complexes. To account for the rate-enhancing effects of the alkali metal cations, mechanistic models have been proposed in which the cation is directly involved in the polymerization process (Fig. 24b). Based on ^31^P NMR spectroscopic studies, the presence of an alkali metal cation in the heterometallic catalysts is suggested to favor the formation of the *cis* isomer (to varying extents), whereas only the *trans* isomer was detected for the mononuclear analogues. It is hypothesized that similar interactions during alkene isomerization also occur in the polymerization reaction. Complementary DFT studies by Ratanasak, Hasegawa, Parasuk, and co-workers indicate that the alkali metal cation is directly involved in the *cis*/*trans* isomerization step preceding the first ethylene insertion. For both elementary steps, the calculated activation barriers are lowest for Li^+^ and increase from Li^+^ to Cs^+^, consistent with the experimentally observed rate trend (Fig. 24a).^144^

**Fig. 24:**
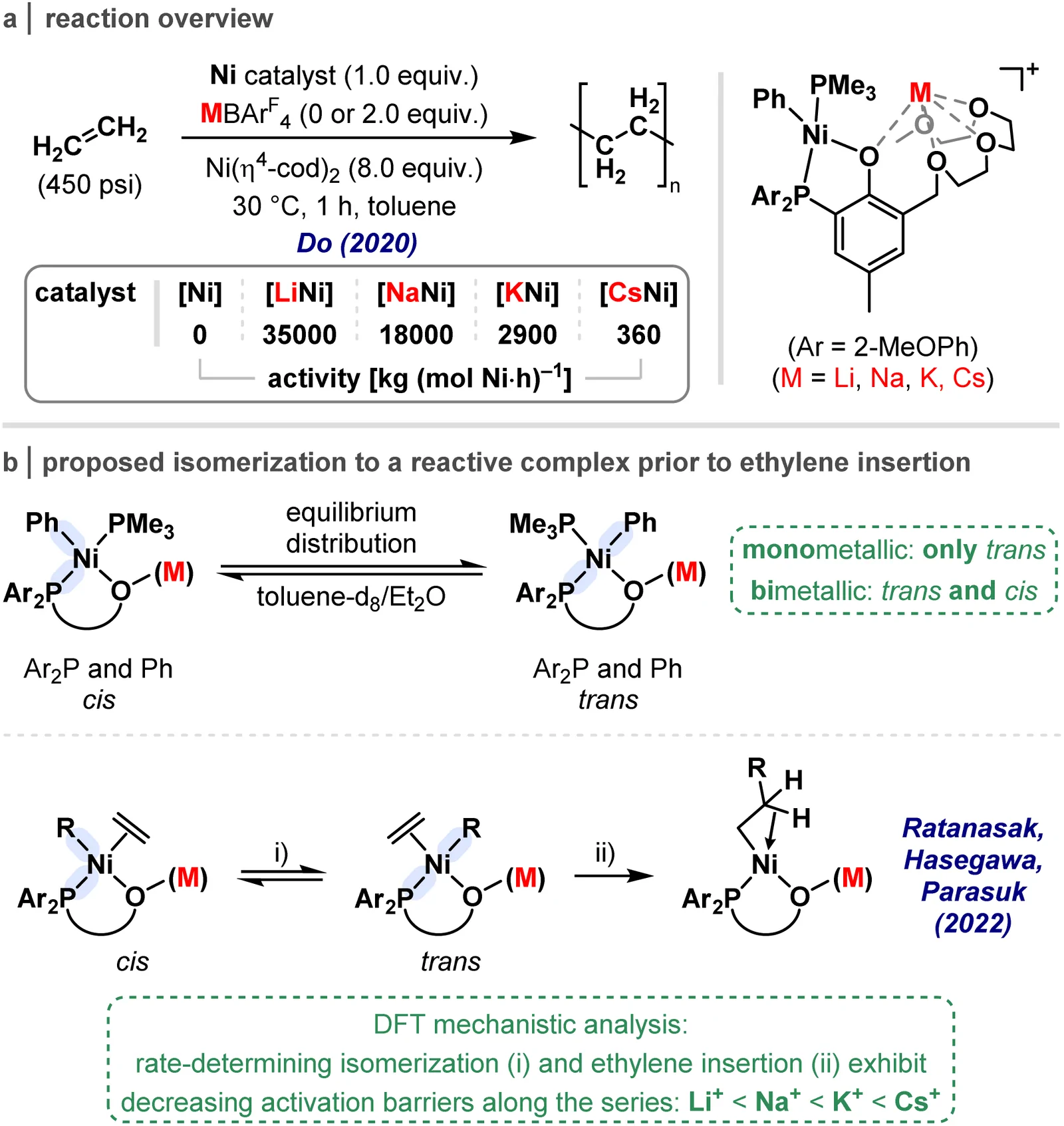
(a) Polymerization of ethylene using alkali metal cation-responsive nickel phenoxyphosphine catalysts. (b) Proposed isomerization to a reactive complex prior to ethylene insertion and complementary DFT investigations.

## 3 Conclusions and outlook

The examples highlighted in this perspective collectively demonstrate that combining s-block and d-block metals creates synergistic activation modes that surpass the intrinsic capabilities of homometallic complexes. The heterometallic complexes discussed in this article enable substrate binding and activation at both metal sites, bond polarization, and concerted activation pathways that depend on the distinct yet synergistic reactivities of the two metal centers. In practice, this synergistic behavior is achieved through different strategies, such as contact ion pairing, preorganized ligand frameworks, and interactions between molecular complexes and metal surfaces. So far, systems based on contact ion pairing have mainly been used in hydrogenation, hydrofunctionalization, and cross-coupling reactions, where cation-dependent reactivity is evident in hydride transfer and dihydrogen activation, as well as in bond activation, such as metalation of C–H, C–O, and C–C bonds. Assemblies that combine homogeneous and heterogeneous catalysts are primarily limited to hydrogenation. By comparison, preorganized heterometallic architectures are primarily applied in alkene isomerization, alkene polymerization, and RO(CO)P. In this case, the synergistic behavior of the s-block and d-block metals is utilized in substrate gating and the selective coordination of alkene, alkoxide, carbonate, and epoxide substrates to polymerization catalysts. In all systems, variations in Lewis acidity, solvation dynamics, and ionic radius of the s-block metal cation can significantly influence reaction rate and selectivity. These factors provide a conceptual basis for establishing quantitative structure–activity relationships, as demonstrated in polymerization catalysts, in which catalytic performance correlates with s-block Lewis acidity (Fig. 22). The interplay of mechanistic analysis, ligand design, and computation is essential for understanding the roles of ion pairing, electrostatic interactions, or dynamic coordination environments in many of the examples discussed here.

Several potential future directions emerge from the insights outlined in this perspective. First, quantitative analyses of synergistic effects in s-block/d-block catalysts are largely absent from the literature. While isolated correlations between catalytic performance and s-block metal Lewis acidity have already been identified in polymerization catalysis (Fig. 22), systematic studies remain scarce for other reaction types. Identifying robust descriptors, such as Lewis acidity, ion pairing strength and solvation energies, will be essential for a more detailed, quantitative understanding of catalytic performance and the underlying synergistic interactions across different reaction classes.

Second, the range of s-block/d-block combinations explored to date remains remarkably limited considering the large number of possible metal pairings. Most studies have focused on alkali metal/late transition metal combinations, whereas alkaline earth metal systems, particularly those involving magnesium, remain comparatively underexplored despite their prominent role in several of the most active catalysts discussed herein. The recent emergence of heterometallic beryllium complexes further highlights opportunities for accessing unusual electronic structures and reactivity modes through highly Lewis acidic s-block fragments. Exploration of new combinations involving alkali metals, alkaline earth metals, and early as well as late transition metals will likely uncover new activation pathways.

Third, the increasing success of cooperative interfaces between homogeneous and heterogeneous catalysts indicates that merging these two types offers a promising research direction, blending the selectivity of molecular catalysts with the robustness of heterogeneous catalysts. While most current applications focus on hydrogenation, applying this approach to a wider range of hydrofunctionalization reactions and other bond activation processes could be very attractive.

Another promising direction concerns asymmetric catalysis. The ability of alkali metal and alkaline earth metal cations to organize substrates through electrostatic interactions, ion pairing, and secondary coordination sphere effects offers opportunities for developing enantioselective heterometallic catalysts in which stereocontrol arises from chiral countercations. Indeed, chiral countercation directed catalysis is well established in organic synthesis and has recently begun to emerge in d-block metal systems paired with chiral organic cations.^145–151^ Therefore, similar approaches using chiral s-block metal cations in combination with catalytically active transition metalate anions could provide a valuable complement to established transition metal-based asymmetric catalysis.

Finally, polymerization catalysis appears particularly well positioned for future advances. Despite clear evidence for strong cation effects on catalyst activity and polymer properties, alkene polymerization by heterometallic catalysts remains less developed than that of RO(CO)P systems. Future studies should therefore focus on extending these concepts beyond ethylene polymerization to polar olefins, diene polymerization, and other monomer classes.

In summary, the work reviewed here points to several intriguing future research goals: (i) quantifying synergistic reactivity, (ii) broadening the range of available s-block and transition metal combinations, (iii) engineering ion pairing and secondary interactions to enhance selectivity, (iv) developing innovative cooperative systems that integrate homogeneous and heterogeneous catalysis, and (v) exploring heterometallic systems for new applications such as asymmetric catalysis and advanced polymerization. As knowledge advances in these areas, heterometallic catalysts are expected to provide increasingly sophisticated and selective means of activating substrates, thereby firmly establishing s-block/d-block cooperation as a versatile concept for catalyst design.

## Author contributions

Martin Gawron conceptualization, data curation, writing – original draft, writing – review and editing; Robert Wolf conceptualization, funding acquisition, supervision, writing – review and editing.

## Conflicts of interest

There are no conflicts to declare.

## Data Availability

No primary research results have been included, and no new data were generated or analyzed as part of this review.
